# Systematic literature review of the somatic comorbidities experienced by adults with phenylketonuria

**DOI:** 10.1186/s13023-024-03203-z

**Published:** 2024-08-12

**Authors:** Kaleigh B. Whitehall, Sarah Rose, Gillian E. Clague, Kirsten K. Ahring, Deborah A. Bilder, Cary O. Harding, Álvaro Hermida, Anita Inwood, Nicola Longo, François Maillot, Ania C. Muntau, André L. S. Pessoa, Júlio C. Rocha, Fran Rohr, Serap Sivri, Jack Said, Sheun Oshinbolu, Gillian C. Sibbring

**Affiliations:** 1 BioMarin Pharmaceutical Inc., 105 Digital Drive, Novato, CA 94949 USA; 2 Clinic for PKU, Copenhagen University Hospital, Copenhagen, Denmark; 3 Huntsman Mental Health Institute, University of Utah, Salt Lake City, UT USA; 4 Oregon Health & Science University, Portland, OR USA; 5 University of Santiago de Compostela, Santiago de Compostela, Spain; 6 Queensland Lifespan Metabolic Medicine Service, Queensland Children’s Hospital, South Brisbane, QLD Australia; 7 University of Utah School of Medicine, Salt Lake City, UT USA; 8 Internal Medicine Department and Reference Center for Inherited Metabolic Disease, and the University of Tours, Tours, France; 9 University Children’s Hospital, University Medical Center Hamburg-Eppendorf, Hamburg, Germany; 10 Federal University of Ceará - UFC, Fortaleza, Ceará, Brazil; 11 Hospital Infantil Albert Sabin, Fortaleza, Ceará, Brazil; 12 NOVA Medical School/Faculdade de Ciências Médicas (NMS/FCM), Universidade NOVA de Lisboa, Lisbon, Portugal; 13 Reference Centre of Inherited Metabolic Diseases (RC-IMD), Centro Hospitalar Universitário de Lisboa Central, Lisbon, Portugal; 14 Nutrition & Metabolism, CINTESIS, NOVA Medical School, Faculdade de Ciências Médicas, Universidade NOVA de Lisboa, Lisbon, Portugal; 15 Met Ed Consultants, Boulder, CO USA; 16 Hacettepe University, Ankara, Turkey; 17 Prime, Knutsford, UK; 18 Prime, London, UK

**Keywords:** Phenylketonuria, Phenylalanine hydroxylase, Phenylalanine, Comorbidity, Diet, Disease burden, Systematic review, Adherence, Nutritional status

## Abstract

**Background:**

Phenylketonuria (PKU) is an inborn error of phenylalanine (Phe) metabolism that, if untreated, causes Phe accumulation in the brain leading to neurophysiologic alterations and poor outcomes. Lifelong management centers on dietary Phe restriction, yet long-term complete metabolic control is unachievable for many adults. High blood Phe levels or chronic Phe and intact protein restriction in the diet may lead to somatic comorbidities. A systematic literature review was conducted to evaluate somatic comorbidities experienced by adults with PKU.

**Methods:**

Clinical and observational studies reporting somatic comorbidities experienced by individuals with PKU aged ≥ 16 years (or classified as adults) evaluating a Phe-restricted diet with or without pharmacologic therapy versus no therapeutic intervention (including healthy controls), or pharmacologic therapy versus a Phe-restricted diet alone, were identified. PubMed® was searched (February 1, 2022 and updated November 1, 2023), using a pre-defined search strategy, followed by two-stage screening and data extraction. Included studies were grouped by PKU population comparison.

**Results:**

1185 records were screened; 51 studies across 12,602 individuals were extracted. Bone-related abnormalities were the most reported outcome (*n* = 21); several outcome measures were used. Original study groupings included: Phe-restricted diet versus healthy controls or reference values (*n* = 40); treatment-adherent versus those non-adherent (*n* = 12). Additional groups added as part of a protocol amendment included: different Phe-restricted diets (*n* = 4); severe versus less severe disease (*n* = 5). Vote counting indicated a higher burden of ≥ 1 comorbidity (or outcome measure) for the Phe-restricted diet group by 37 of 38 studies included in the analysis of Phe-restricted diet versus healthy controls; higher burden in healthy controls was reported in 12 studies. Vote counting was similar between those treatment adherent (*n* = 7) versus non-adherent (*n* = 10).

**Conclusions:**

Adults with PKU have a higher comorbidity burden than a non-PKU population. More robust studies are needed to better understand the relationship between effective metabolic control and comorbidity burden, using consistent outcome measures.

This SLR was supported by BioMarin Pharmaceutical Inc., Novato, CA, and is registered with the Research Registry (reviewregistry1476).

**Supplementary Information:**

The online version contains supplementary material available at 10.1186/s13023-024-03203-z.

## Background

Phenylketonuria (PKU), as a colloquial term for phenylalanine hydroxylase (PAH) deficiency (OMIM# 261600), is an autosomal recessive inborn error of amino acid metabolism. PKU is caused by pathogenic variants in the gene encoding PAH, impairing enzyme function such that PAH cannot metabolize phenylalanine (Phe) to tyrosine normally. Phe accumulates in the blood, crossing the blood–brain barrier at high concentrations with toxic effects. Phe also competes with other large neutral amino acids for transport across the blood–brain barrier by the L-type amino acid transporter 1 (LAT1); high concentrations of Phe may block transport of other LAT1 substrates into the brain, including tyrosine and tryptophan, important for neurotransmission [1]. If left untreated, PKU is associated with poor neurologic, neurocognitive, and neuropsychiatric outcomes [1, 2].

Recognized as exhibiting a spectrum of severity, the most severe form of PKU is often referred to as classical PKU (cPKU) and is defined as little or no PAH activity and untreated blood Phe levels typically > 1200 µmol/L at the time of diagnosis (normal blood Phe level is 50–110 µmol/L). An individual’s specific genetic variation determines the degree of PAH activity; variants only partially inhibiting PAH activity result in a milder form of PKU or mild hyperphenylalaninemia (HPA) [2].

The goal of treating PKU is to achieve and maintain appropriate blood Phe levels recommended by the United States and European guidelines [3, 4]. To control blood Phe levels, individuals with PKU are placed on a lifelong prescribed medical intervention termed medical nutrition therapy (MNT) [4], which involves severely restricting the natural intake of protein and replacing it with a Phe-free, amino acid-based medical food to supplement the reduced protein intake, and provide a source of energy and other nutrients. Supplements might include modified low-protein foods and Phe-free medical food beverages, Phe-free amino acid mixture, medical foods derived from glycomacropeptide, and protein substitutes [4].

Importantly, studies have shown that not all patients, including adolescents and adults, are able to achieve blood Phe levels within guideline-recommended target ranges [5, 6]. Even with active management, blood Phe levels may remain uncontrolled, especially as patients age. An online survey conducted in the United States estimated that 67% of adults with PKU had blood Phe levels in excess of the upper limit of the American College of Medical Genetics target levels [7]. Patients with cPKU have the most difficulty controlling blood Phe levels with MNT, and control is considered suboptimal when compared with patients with mild PKU [6].

Pharmaceutical intervention with sapropterin dihydrochloride (KUVAN®; BioMarin Pharmaceutical Inc., Novato, CA, USA), a derivative of the PAH cofactor, tetrahydrobiopterin (BH4), may be used in conjunction with a Phe-restricted diet, for individuals who are deemed responsive. For adults (or patients ≥ 16 years old outside of the United States) with uncontrolled blood Phe levels despite existing management, pegvaliase (PALYNZIQ®; BioMarin Pharmaceutical Inc., Novato, CA, USA), a PAH substitute [8, 9], may be an option to achieve appropriate blood Phe levels, without requiring patients to maintain a Phe-restricted diet [10].

Lifelong treatment of PKU is recommended by guidelines [3, 4]. Early intervention prevents the severe and irreversible intellectual impairment caused by elevated blood Phe levels in childhood and adolescence [11], but adherence to dietary restrictions is challenging, and the number of patients achieving target blood Phe levels tends to diminish with age [2, 7]. Uncontrolled Phe levels are also associated with adverse neurocognitive and neuropsychiatric outcomes in adults [12–14]. Meta-analysis of cognitive function in adults with PKU has shown impairment in reasoning, visuo-spatial attention speed, sustained attention, and visuo-motor control, despite early initiation of treatment [14], and meta-analysis of neuropsychiatric symptoms in adults with PKU has shown that inattention, hyperactivity, depression, and anxiety exceed general population estimates [13]. Neurocognitive and neuropsychiatric symptoms associated with Phe accumulation may make it more difficult for patients to adhere to dietary restriction, which in turn can lead to poor blood Phe control and worsening of symptoms [4].

An emerging body of literature suggests that the impact of PKU on an individual’s health may extend beyond symptoms of a neurocognitive and neuropsychiatric nature. Comorbidities across various organ systems have been reported in adults with PKU, with health insurance claims-based studies suggesting a higher prevalence of somatic comorbidities compared with a general population [15, 16].

High blood Phe levels may impact biologic mechanisms that are related to increased risk of comorbid conditions such as obesity, renal disease, metabolic dysfunction, and cardiovascular complications [16]. Due to the impact on different organ systems, the etiology is complex and multifactorial [17]. Retrospective analysis of insurance claims data has enabled researchers to generate hypotheses for development of certain comorbidities based on their knowledge of PKU and the associated dietary management [15, 16]. Better understanding of the etiology of somatic comorbidities associated with PKU and identification of factors other than high blood Phe that may be preventable or amenable to treatment, together with effective metabolic control, could aid in reducing the burden of illness and healthcare costs. However, the first step is to investigate differences in somatic comorbidity burden, not only between adults with PKU and the general population but also among adults with PKU receiving different therapeutic interventions, those adherent to treatment or not, and with different disease severity.

A systematic literature review (SLR) has been conducted to evaluate the published evidence on the somatic comorbidities experienced by adults with PKU. The SLR aims to further characterize the physical health burden of PKU and provide insight into the impact of differences in therapeutic interventions, adherence to treatment, and differences in disease severity on the somatic comorbidity burden.

## Materials and methods

The SLR is registered with the Research Registry with the unique identifying number review registry 1476 and is reported in accordance with the Preferred Reporting Items for Systematic Reviews and Meta-Analyses (PRISMA) and Synthesis Without Meta-analysis (SWiM) guidelines [18, 19].

### Eligibility criteria

Eligibility criteria for the inclusion of studies to address the research question were established using the Population, Intervention, Comparator, Outcome, Study design (PICOS) framework (Table 1).

**Table 1:** Inclusion criteria established using the PICOS framework

	**Inclusion criteria**	**Exclusion criteria**
Population	Adults (aged ≥ 16 years, or as defined by the study) with confirmed PKU or described as having PKU	Children aged < 16 years^a^
Intervention	• Phe-restricted diet/MNT: ◦ Protein substitutes, including: ▪ infant protein substitutes (powder) ▪ infant protein substitutes (liquid) ▪ powdered weaning protein substitutes ▪ semi-solid weaning protein substitutes ▪ powdered protein substitutes ▪ liquid protein substitutes ▪ protein substitute tablets ▪ protein substitute bars ◦ Low-protein foods, including: ▪ fruits and vegetables ▪ fats ▪ starches ▪ vegan cheese ▪ vegetarian jelly/agar (gelatin free) ▪ low-protein special foods: low-protein breads, flour mixes, pizza bases, pasta, biscuits, and egg replacers containing < 25 mg Phe/100 g ▪ herbs/spices ▪ drinks ▪ low-protein/low-Phe special milk ▪ plant milk • Sapropterin dihydrochloride or pegvaliase	
Comparator	• Phe-restricted diet/MNT (with or without pharmacologic therapy) comparator: ◦ No therapeutic intervention (not receiving Phe-restricted diet/MNT) including healthy controls or a reference population • Sapropterin dihydrochloride or pegvaliase comparator: ◦ Protein substitutes and/or low-protein foods	
Outcomes^b^	• The prevalence of different somatic comorbidities in patients with PKU receiving Phe-restricted diet/MNT, and/or sapropterin dihydrochloride or pegvaliase compared with: ◦ Healthy controls ◦ The general population (including standard reference values) ◦ Patients with PKU not receiving any form of therapeutic intervention ◦ Patients with PKU who did not adhere to treatment ◦ Patients with PKU who interrupted/ discontinued treatment • The severity of different somatic comorbidities in patients with PKU receiving Phe-restricted diet/MNT, and/or sapropterin dihydrochloride or pegvaliase compared with all groups listed above • The prevalence of different somatic comorbidities in patients across the PKU disease spectrum receiving Phe-restricted diet/MNT, and/or sapropterin dihydrochloride or pegvaliase • The severity of different somatic comorbidities in patients across the PKU disease spectrum receiving Phe-restricted diet/MNT, and/or sapropterin dihydrochloride or pegvaliase	
Study design/ publication type	• Randomized controlled trials • Single-arm clinical trials • Cohort studies (prospective and retrospective) • Cross-sectional studies and surveys	• Systematic reviews and meta-analyses^c^ • Narrative (non-systematic) review articles • Animal studies • In vitro studies • Letters, editorials, and commentaries • Guidelines and best practice • Congress abstracts • Non-peer-reviewed articles
Language of publication	English	
Date of publication	Up to November 1, 2023	
Countries	All	-

*Abbreviations: MNT* medical nutrition therapy, *Phe* phenylalanine, *PICOS* Population, Intervention, Comparator, Outcome, Study type, *PKU* phenylketonuria

^a^Studies reporting on an exclusive population of children < 16 years, were excluded, but studies reporting a mixed-age population were included

^b^Studies comparing different Phe-restricted diets and those comparing populations of individuals with more severe disease and those with less severe disease were also considered eligible in a protocol amendment that was made during the full-text review stage

^c^Systematic reviews and meta-analyses were used for backwards citation searching but were not incorporated in the data synthesis

Peer-reviewed observational studies (cohort, case–control, cross-sectional, surveys) and clinical trials in individuals ≥ 16 years of age (or defined as adults by the study) either confirmed or described as having PKU were included. The cut-off for adult age was chosen to be ≥ 16 years because adolescent age differs globally. Eligible studies were those evaluating a Phe-restricted diet with or without pharmacologic therapy (sapropterin dihydrochloride or pegvaliase) versus no form of therapeutic intervention (including healthy controls or reference values); or pharmacologic therapy versus a Phe-restricted diet alone. Studies comparing different Phe-restricted diets (e.g., different modified low-protein foods and Phe-free medical food beverages, Phe-free amino acid mixture, medical foods derived from glycomacropeptide) and those comparing populations of individuals with more severe disease and those with less severe disease, were also considered eligible in a protocol amendment that was made during the full-text review stage. Outcomes of interest were defined in the study protocol as the prevalence and/or severity of somatic comorbidities in individuals with PKU, but in practice, any measure of somatic comorbidity experienced by individuals with PKU in eligible studies was considered for inclusion.

Studies carried out exclusively in a population of individuals identified as children or adolescents were excluded; however, otherwise eligible studies with mixed-age populations were included whether or not results were presented separately for adults. Non-human studies and in vitro studies, single-cohort studies in individuals with PKU who were untreated or in individuals with PKU on Phe-restricted diet that were not compared with a healthy control population or reference values, or in individuals with PKU treated with pharmacologic therapy who were not compared with those on Phe-restricted diet alone, were excluded. Secondary literature sources, including narrative review articles, letters, editorials, and commentaries were also excluded, as were therapy recommendations, clinical guidelines, congress abstracts, and non-peer-reviewed literature.

### Information sources and search strategy

Literature was retrieved via the PubMed® interface. No date restrictions were applied to the search, thus publications in English from MEDLINE earliest coverage to November 1, 2023 were included (search conducted on February 1, 2022 and updated on November 1, 2023) [20]. A pre-defined systematic search strategy (Additional file 1: Table S1) was designed to identify relevant records; the search string included a combination of free text and Medical Subject Headings (MeSH) search terms based on the inclusion and exclusion criteria documented in Table 1. To maximize the identification of relevant articles, the search string included general terms for comorbidity and burden, as well as specific somatic comorbidity types guided by the results of a previous (unpublished) literature review that was available to authors designing the study.

Backwards citation searching was employed to identify additional papers of interest in the reference lists of relevant systematic reviews and meta-analyses that were retrieved as part of the systematic literature search and search update. Duplicate records were removed during screening.

### Selection process

Study screening was carried out by a small team of reviewers to identify records eligible for data extraction according to the PICOS framework. A small number of records (*n* = 10) were screened independently by the reviewing team in a pilot phase, and results compared and discussed to assess concordance of eligibility decisions and ensure relevance and utility of the inclusion and exclusion criteria used to screen records.

A two-stage screening process was then applied to identify records eligible for data extraction according to the inclusion and exclusion criteria. Records were screened once by title and abstract and selected for full-text review if they met all inclusion criteria or if it was unclear whether all inclusion criteria were met; records were only rejected if it was clear that at least one of the inclusion criteria was not met (termed positive exclusion methodology). Records considered potentially eligible were screened once by full text to confirm eligibility for data extraction. Concordance of eligibility decisions was assessed at both screening stages, whereby 10% of records underwent a second independent screen and any discrepancies in first and second reviewer opinion were discussed with a third reviewer to achieve a consensus decision.

Screening was carried out within the DistillerSR Inc. (Ottawa, ON, Canada) workflow management software and used to view records for review, indicate conflicts between and record reviewers’ decisions, including reasons for exclusion. The continuous artificial intelligence reprioritization feature was utilized to continuously re-order the screening of records based on previous screening decisions.

### Data collection and data outcomes

Data extraction from eligible studies was conducted by one reviewer into a pre-designed data extraction spreadsheet (Microsoft Excel®; Microsoft Corporation, Redmond, WA, USA). All data that related to outcomes of interest, including any measure or description of any somatic comorbidities experienced by individuals with PKU, were extracted, as well as other data items including study design, geographic coverage, year of publication, main study conclusions and limitations. Statistical comparisons between groups were also recorded, when available. Studies reporting insufficient data to satisfy inclusion criteria (e.g., data were not reported separately for group of interest) were rejected. Extracted data were checked for accuracy by an independent reviewer.

### Grouping studies for synthesis

Studies were grouped by PKU population to allow synthesis of data according to the populations identified in the research question (i.e., those on a Phe-restricted diet with or without pharmacologic therapy [sapropterin dihydrochloride or pegvaliase] versus healthy controls or reference values; those on a Phe-restricted diet who were adherent versus non-adherent; those on different Phe-restricted diets; those on a Phe-restricted diet with more severe PKU versus those with HPA or less severe PKU).

Studies were then grouped according to comorbidity type (abdominal or pelvic pain, bone-related abnormalities, cancer, cardiovascular outcomes, chronic obstructive pulmonary disease [COPD]/asthma, dermatologic disorders, diabetes, gastrointestinal disorders, hypertension, migraine/headache, musculoskeletal outcomes, nutritional outcomes, overweight/obesity, or other) to allow synthesis of data by specific comorbidity type.

### Data synthesis

The breadth of measures and numbers of studies in each population grouping that reported outcome measures for the same comorbidity type were analyzed to identify appropriate methods for data synthesis (e.g., meta-analysis or synthesis without meta-analysis) to determine intervention effects.

Bone mineral density (BMD) Z-scores, where a low BMD Z-score is considered an indicator of bone-related abnormality, was the only outcome measure considered appropriate for meta-analysis of effect estimates due to sufficiency and homogeneity in clinical outcomes, methodological approach, and statistics reported, and this analysis is reported separately. Meta-analysis of effect estimates was not considered appropriate for the other somatic, comorbidity types due to extensive heterogeneity in clinical outcomes used, the definitions of clinical outcomes used, how the clinical outcome was measured, and study design including interventions and comparators. Vote counting was considered an acceptable alternative method given it allows the direction of effect to be determined using all available evidence, for example, even when there is no consistent effect measure or data reported across studies.

Vote counting was conducted according to the methods described in the Cochrane handbook and reported according to the SWiM guidelines [19, 21]. A standardized binary metric was created by allocating votes to individual studies according to the direction of a higher comorbidity burden (i.e., either a higher burden in the direction of the ‘intervention’ [for example, individuals with PKU on a Phe-restricted diet with or without pharmacologic therapy] or a higher burden in the direction of the ‘comparator’ [for example, healthy controls or reference values]), regardless of the statistical significance clinical relevance of differences between the groups. The number of votes allocated to the intervention population was then compared with the number allocated to the comparator population, to determine the direction of effect, and was visualized using an effect direction plot, in line with guidance from the Cochrane handbook [21]. Studies were prioritized for data synthesis based on directness in relation to the research question and availability of data. No assessment of certainty of the evidence was undertaken given it is difficult to assess consistency of effects when vote counting is undertaken.

## Results

### Study selection and characteristics

**Fig. 1:**
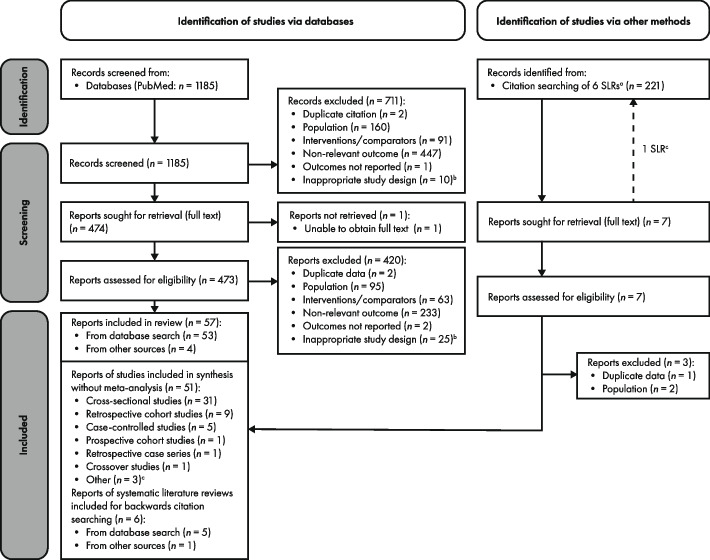
PRISMA diagram showing article selection process. Articles were excluded on a hierarchical basis, in the order that questions were asked (i.e., if the answer to the first question was no, this was given as the main reason for exclusion, but articles may have met or not met other criteria). Abbreviation: SLR, systematic literature review. ^a^Five systematic reviews were identified via the database search and used for backwards citation-searching only plus one additional systematic review identified via backwards citation-searching that was then used for further backwards citation-searching; ^b^Includes studies that did not present outcomes in a meaningful way which answered one or more of the pre-specified research questions; ^c^Other studies include open interventional trials, pooled analyses, and cost analyses

The PRISMA flow diagram (Fig. 1) shows the results of the study selection process. The PubMed® search identified 1185 unique records. Subsequently, 473 records were considered potentially eligible for inclusion and 53 studies were confirmed as eligible for inclusion. Five included studies from the PubMed® search were SLRs and used for backwards citation searching only, revealing five additional papers of interest, of which two were confirmed as eligible for inclusion. One of these studies was an SLR and backwards citation-searching revealed two publications of interest, of which only one met inclusion criteria. Overall, a total of 57 studies met the PICOS criteria. Data were extracted from 51 studies spanning 12,602 individuals (excluding the six SLRs) and included in the synthesis. Reasons for exclusion of studies at each stage of the selection process are listed in Fig. 1.

Of the 51 studies included in the synthesis, most were of observational design, including cross-sectional studies (*n* = 31, 61%), retrospective cohort studies (*n* = 9, 18%) and case-controlled studies (*n* = 5, 10%) (Additional file 2: Figure S1). Most studies were conducted in European countries (*n* = 40) and/or in North America (*n* = 6) (Additional file 3: Figure S2).

**Fig. 2:**
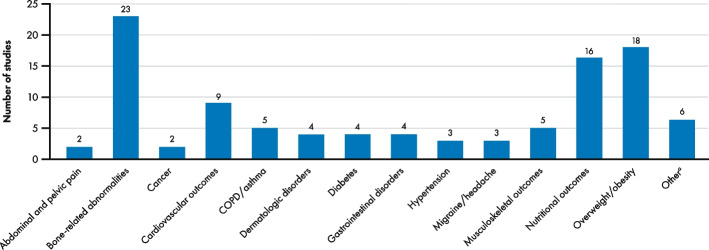
Distribution of studies by comorbidity type. Abbreviations: COPD, chronic obstructive pulmonary disease. ^a^Other comorbidities include: acute upper respiratory infections of multiple and unspecified sites; allergic and chronic rhinitis; anemia; adverse events, not elsewhere classified; calculus of kidneys; Charlson Comorbidity Index score; chronic kidney disease; congenital deformities of feet; dizziness and giddiness; dorsalgia; esophageal disorders; gallbladder disease; grip force; gynecological symptoms; hypothyroidism; menopausal and other perimenopausal disorders; metabolic syndrome; ophthalmological symptoms; other disorders of the urinary system; other hypothyroidism; other non-inflammatory disorders of the vagina; other non-toxic goiter; other and unspecified dorsopathies; other and unspecified soft tissue disorders; otolaryngological symptoms; refraction and accommodation disorders; renal insufficiency with hypertension; renal insufficiency without hypertension; thyroid function; upper respiratory traction infection; varicose veins of lower extremities; vasomotor and allergic rhinitis

More than 13 different comorbidity types were reported across the 51 studies; bone-related abnormalities were the most reported (*n* = 23), followed by overweight/obesity (*n* = 18), nutritional outcomes (*n* = 16), and cardiovascular outcomes (*n* = 9) (Fig. 2). Migraine/headache and cancer were reported in a consistent manner across the studies, whereas outcome measures for bone-related abnormalities, cardiovascular outcomes, and dermatologic disorders were reported with a high degree of inconsistency resulting in heterogeneity. One study used the Charlson Comorbidity Index (CCI) to report multiple comorbidities [22].

### Data synthesis

Vote counting was used to determine the direction of comorbidity burden in two PKU population comparisons: those on a Phe-restricted diet with or without pharmacologic therapy versus healthy controls or reference values, and those on a Phe-restricted diet who were treatment-adherent versus a non-adherent population. For all other PKU population comparisons, data synthesis by vote counting was not feasible due to a low number of studies.

#### Individuals with PKU on a Phe-restricted diet with or without pharmacologic therapy versus healthy controls or reference values

Of the 40 studies comparing individuals with PKU on a Phe-restricted diet, with or without pharmacologic therapy, with healthy controls or reference values (Table 2), two studies were excluded from the vote counting because it was not possible to confirm treatment with a Phe-restricted diet in the full study population [16, 23].

**Table 2:** Summary of characteristics of studies (*n* = 40) in individuals with PKU on a Phe-restricted diet versus healthy controls or reference values

Study	Study location	Study design	Age range (years)	Study size, intervention(s), and comparator(s)	Comorbidity types reported	Burden of comorbidity type
Azabdaftari et al. 2019 [43]	Europe (Germany)	Case-controlled study	Adults 18–47	• 23 individuals with PKU on a Phe-restricted diet • 28 healthy controls	Cardiovascular outcomes	**Significantly higher resting heart rate (*p* = 0.001) and systolic blood pressure (*p* = 0.012) in individuals with PKU compared with controls**
					Overweight/obesity	**Significantly higher BMI compared with healthy controls (*p* < 0.0001),** with a mean BMI of 27.6 (5.4) kg/m^2^ versus 23.4 (6.4) kg/m^2^: 48% had bodyweight within normal range, 19% were pre-obese, 33% were obese
Bilder et al. 2017 [23]	North America (USA)	Retrospective cohort study	Adults 20–NR	• 3714 individuals with PKU (unclear whether individuals were on a Phe-restricted diet) • 22,726 healthy controls	Migraine/headache	**Significantly higher prevalence of migraine/headache in individuals with PKU compared with controls (*p* < 0.05)**
Burton et al. 2018 [16]	North America (USA)	Case-controlled study	Adults 20–NR	• 3691 individuals with PKU (unclear whether individuals were on a Phe-restricted diet) • 18,455 age-matched controls	Overweight/obesity	**Significantly higher prevalence ratio of overweight individuals with PKU compared with controls (*p* < 0.0001)**
					Bone-related abnormalities	**Significantly higher prevalence ratio of individuals with PKU with osteoporosis compared with controls (*p* < 0.001)**
					Gastrointestinal disorders	**Significantly higher prevalence ratio of individuals with PKU with GERD (*p* < 0.001); gastritis and esophagitis (*p* < 0.001) compared with controls**
					COPD/asthma	**Significantly higher prevalence ratio of individuals with PKU with asthma compared with controls (*p* < 0.001)**
					Dermatologic disorders	**Significantly higher prevalence ratio of individuals with PKU with urticaria (p ≤ 0.001); dermatitis and eczema (*p* < 0.001) compared with controls**
					Other	**Significantly higher prevalence ratio of other comorbidities in individuals with PKU compared with controls (*p* < 0.001): renal insufficiency with hypertension; renal insufficiency without hypertension; calculus of kidneys; esophageal disorders; anemia; gallbladder disease; allergic and chronic rhinitis**
Carson et al. 1990 [34]	Europe (Northern Ireland)	Cross-sectional study	Adults 19–34	• 11 individuals with PKU on a Phe-restricted diet • General population	Bone-related abnormalities	**There was a significant reduction in trabecular bone mineral content in individuals with PKU compared with the general population (*p* < 0.001)** During childhood, six of the 11 individuals gave a history of fracture
Charrière et al. 2023 [22]	Europe (France)	Retrospective cohort study	Adults 16–NR	• Assessed for CCI and predetermined comorbidities: • 2175 individuals with PKU (1528 early diagnosed comparison); 28.6% on dietary amino acid supplement, 5.0% on sapropterin dihydrochloride • 10,743 matched controls (7514 early diagnosed comparison) • Assessed for top 50 inpatient ICD-10 codes: • 1911^a^ individuals with PKU (1301^a^ early diagnosed comparison); 40.4% on dietary amino acid supplement, 7.0% on sapropterin dihydrochloride • 9505 matched controls (6457 early diagnosed comparison)	Other	**Significantly higher mean CCI score for overall PKU population versus non-PKU controls (*p* < 0.0001),** although low in both groups: 0.4 (SD 1.2) versus 0.2 (SD 0.7) The mean CCI score was 0.0 both for individuals with early diagnosed PKU and their controls (*p* = 0.4897) **Significantly higher frequency of chronic kidney disease (*p* < 0.0001), sleep disorders (*p* < 0.0001), SIRS (*p* = 0.0033), respiratory failure (*p* < 0.0001), malaise and fatigue (*p* < 0.0001), and other functional implants (*p* = 0.0290)** but no significant difference in senile cataract (*p* = 0.5154) and embedded and impacted teeth (*p* = 0.3248) in overall PKU population versus non-PKU controls For the early diagnosed subgroup comparison, **only sleep disorders remained significantly higher versus non-PKU controls (*p* < 0.0089)**; chronic kidney disease (*p* = 0.3331), SIRS (*p* = 1.0), respiratory failure (*p* = 0.1982), other functional implants (*p* = 1.0), malaise and fatigue (*p* = 0.1272), and embedded and impacted teeth (*p* = 0.3647)
					Bone-related abnormalities	**Significantly higher percentage of individuals with osteoporosis in overall PKU population versus controls (28.7% versus 19.8%, *p* < 0.0001)** **Percentages with osteoporosis were lower in the early diagnosed subgroup (0.3% versus 0.01%) but statistical significance of the comparison with controls was maintained (*p* = 0.0035)**
					Cardiovascular	**Significantly higher percentage of individuals with ischemic heart diseases (*p* < 0.0001), heart failure (*p* = 0.0002), atrial fibrillation and flutter (*p* = 0.0018), and cardiac and vascular implants and grafts (*p* < 0.0001) in overall PKU population versus controls** No cardiovascular outcomes were included in the assessment of the top 50 inpatient ICD-10 codes for the early diagnosed subgroup comparison
					Hypertension	**Significantly higher percentage of individuals with hypertension in overall PKU population versus controls (20.9% versus 17.0%, *p* < 0.0001) and in the early diagnosed group versus controls (4.0% versus 2.7%, *p* = 0.0063)**
					Diabetes	**Significantly higher percentage of individuals with diabetes in overall PKU population versus controls (7.8% versus 4.7%, *p* < 0.0001)** The difference between groups for the early-diagnosed subgroup was not statistically significant (*p* = 1.0)
					Overweight/obesity	**Significantly higher percentage of individuals with obesity in overall PKU population versus controls (4.2% versus 1.3%, *p* < 0.0001), which was maintained in the early diagnosed subgroup comparison**
					Nutritional outcomes	**Significantly higher percentage of individuals with vitamin D deficiency (*p* = 0.0002) and iron deficiency anemia (*p* < 0.0001) in overall PKU population versus controls** **Vitamin D deficiency was maintained in the early diagnosed subgroup comparison (*p* < 0.0001)** but the difference for iron deficiency anemia was not statistically significant (*p* = 0.5451)
					Gastrointestinal disorders	No significant difference in percentages of individuals with diverticular disease of the intestine (*p* = 0.2698), gastritis and duodenitis (*p* = 0.2505), or constipation (*p* = 0.9421) in overall PKU population versus controls Results for the early diagnosed comparison were similar: diverticular disease of the intestine (*p* = 0.5202), gastritis and duodenitis (*p* = 0.5503), or constipation (*p* = 0.7574)
					COPD/asthma	**Significantly higher percentage of individuals with asthma (*p* = 0.0277) in overall PKU population versus controls;** however, the percentages in each group were the same for the early diagnosed subgroup comparison (*p* = 0.7282)
					Musculoskeletal outcomes	**Significantly higher percentage of individuals with abnormalities of gait and mobility (*p* < 0.0001) in overall PKU population versus controls, which was maintained in the early diagnosed subgroup comparison (*p* < 0.0047)**
					Abdominal and pelvic pain	No significant difference in percentages of individuals with abdominal and pelvic pain (*p* = 0.3887) in overall PKU population versus controls and for the early diagnosed subgroup comparison (*p* = 0.5396)
Choukair et al. 2017 [24]	Europe (Germany)	Cross-sectional study	11–42	• 56 individuals with PKU on a Phe-restricted diet (40 cPKU, 15 mild PKU, and one HPA. Some individuals were not on a Phe-restricted diet: 33 were on a PKU diet supplemented with amino acids; 16 did not follow a diet or take amino acid supplementation; three had amino acid supplementation only; four followed a PKU diet exclusively) • 700 reference population	Bone-related abnormalities	**Significantly lower distal radius (total BMD *p* = 0.0001), cortical thickness (*p* = 0.0001), and SSI (*p* = 0.0001) at proximal radius in individuals with PKU compared with the reference population** Individuals with PKU had a comparable cortical density compared with the reference population (*p* = 0.529)
					Musculoskeletal outcomes	**Significantly lower mean standardized muscle cross-sectional area (*p* < 0.0001) and grip force (*p* = 0.0004) in individuals with PKU compared with the reference population** **Regression slope between SSI and corresponding muscle cross-sectional area was significantly (*p* < 0.0001) less steep in the reference population indicating bone strength in individuals with PKU is not adequately adapted to muscle force**
						Muscle size and performance were preserved in individuals with PKU and regression lines were comparable with the reference population (*p* = 0.369)
Coakley et al. 2016 [25]	North America (USA)	Cross-sectional study	4– ≥ 40	• 88 individuals with PKU on a Phe-restricted diet (15% were on sapropterin dihydrochloride or pegvaliase) • Reference values used for Z-score calculation	Bone-related abnormalities	A total of 18 participants (20.4%) had a TBMD Z-score < –1. In the whole sample, mean TBMD Z-score was -0.326 (SD = 0.112), within normal range of BMD for age (Z-score > –2) There were no significant differences (*p* > 0.05) in BMD Z-score by age group, male: female ratio, BMI category, AV sum category, menstruation status, or blood vitamin D category
Couce et al. 2018 [44]^b^	Europe (Spain)	Cross-sectional study	NR	• 33 individuals with PKU on a Phe-restricted diet • 68 healthy controls	Diabetes	Higher HOMA-IR in individuals with PKU compared with controls
					Overweight/obesity	BMI was above the upper limit in 32 individuals with PKU (38.6%), 75% of whom were overweight and 25% of whom were obese In the control group of 68 healthy cases (age range: 4–49 years), 25% (17 cases: 10 obese and seven overweight) had a BMI above the upper limit
Crujeiras et al. 2015 [45]^c^	Europe (Spain)	Cross-sectional study	0.6–42	• 156 individuals with PKU or mild HPA (unspecified whether all were on a Phe-restricted diet) • 122 individuals with PKU; 18.8% (*n* = 23) treated with BH4 supplementation; 86 with cPKU and 36 with mild or moderate PKU (*n* = 2 and n = 21 treated with BH4 supplementation, respectively) • Reference values used to determine pathologic concentrations of vitamins and minerals	Nutritional outcomes	Percentage of individuals with PKU that had pathologic biochemical values: 34.6% pre-albumin, 7.7% for ferritin, 25% for selenium, 39% for folic acid, and 5.1% for zinc Total protein, calcium, phosphorus, vitamin B12, ferritin, and zinc levels were found to be in the normal range in almost all individuals with PKU No deficiency of folic acid was found in the study cohort, but was found to be above the upper limit in 39% of participants
de Groot et al. 2012 [41]	Europe (The Netherlands)	Cross-sectional study	Adults 20–NR	• 18 individuals with PKU on a Phe-restricted diet • Reference values used for Z-score calculation	Bone-related abnormalities	**BMD was significantly reduced in individuals with PKU compared with reference values (*p* = 0.004) (mean Z-scores of BMD were significantly lower than zero in individuals with PKU)**
Demirdas et al. 2017 [26]	Europe (The Netherlands)	Cross-sectional study	1–39	• 60 individuals with PKU; 76.7% (*n* = 46) on a Phe-restricted diet plus AAM; 16.7% (*n* = 10) on a Phe-restricted diet plus AAM plus BH4; 6.7% (*n* = 4) on BH4 alone • Reference values/general population	Bone-related abnormalities	Lumbar and femoral BMD Z-scores were lower than zero (< –2) in 4.9% and 7.4% of individuals with PKU, respectively Lifetime fracture prevalence was comparable with the general population
					Nutritional outcomes	Blood concentrations of vitamin D2 and D3, and selenium were within (*n* = 52 and *n* = 30, respectively) or below (*n* = 7 and *n* = 29, respectively) the reference range in all individuals with PKU Blood concentrations of magnesium, folate, vitamin B12, and vitamin B6 were above (*n* = 24, *n* = 29, *n* = 11, and *n* = 55, respectively) or within (*n* = 35, *n* = 27, *n* = 47, and *n* = 5, respectively) the reference range in all individuals with PKU Blood concentrations of zinc were above (*n* = 1), within (*n* = 49), and below (*n* = 8) the reference range
Dios-Fuentes et al. 2022 [32]^d^	Europe (Spain)	Retrospective cohort study	Adults 23–38	• 90 individuals with PKU (72 on a Phe-restricted diet, 18 on sapropterin dihydrochloride) • 67 analyzed for lumbar spine BMD and 62 analyzed for femoral neck BMD • 62 early diagnosed (54 cPKU, four moderate PKU, four mild PKU) • 28 late diagnosed (26 cPKU, one moderate PKU, one mild PKU)	Bone-related abnormalities	According to the BMD report in individuals with PKU: 48.5% had normal BMD, 41.2% had osteopenia, and 10.3% had osteoporosis Lumbar spine BMD was normal in 50.7%, showed osteopenia in 38.8%, and osteoporosis in 10.4%; median Z-score –1.1 (range: –1.75, –0.35) Femoral neck BMD was normal in in 64.5%, showed osteopenia in 32.3% and osteoporosis in 3.2%; median Z-score –0.45 (range: –1.1, 0.55) Difference between lumbar spine and femoral neck Z-score was statistically significant (*p* = 0.004)
Greeves et al. 1997 [35]	Europe (UK)	Case-controlled study	0.1–33.6	• 85 individuals with PKU on a Phe-restricted diet • 98 healthy controls	Bone-related abnormalities	**Significantly higher risk of fracture reported in individuals with PKU ≥ 8 years of age whose fracture rate was 2.6 times greater than the rate in controls (*p* = 0.024)**
						No significant difference in the lifetime risk of fractures between individuals with PKU and controls (*p* = 0.23)
Hermida-Ameijeiras et al. 2017 [46]	Europe (Spain)	Cross-sectional study	6–50	• 41 individuals with PKU on a Phe-restricted diet (22 cPKU and 19 mild or moderate PKU). Seven individuals were treated with sapropterin dihydrochloride in addition to dietary treatment • 41 healthy controls	Cardiovascular outcomes	**Individuals with cPKU had a significantly higher pulse-wave velocity (a systemic arterial stiffness marker and predictor of adverse cardiovascular events) compared with controls (*p* < 0.05)**
						Arterial stiffness markers (augmentation pressure *p* = 0.139, augmentation index *p* = 0.426) were increased in individuals with PKU compared with controls
Htun et al. 2015 [47]	Europe (Germany)	Prospective cohort study	NR–38	• 43 individuals with PKU on a Phe-restricted diet • 58 healthy controls	Cardiovascular outcomes	No significant difference in CIMT (*p* = 0.88) and β-stiffness index (*p* = 0.7) between individuals with PKU and controls
Huemer et al. 2008 [48]	Europe (Austria)	Case-controlled study	3.9–20.4	• 37 individuals with PKU on a Phe-restricted diet • 63 healthy controls • Reference values	Nutritional outcomes	**Cobalamin (*p* < 0.0001) and folate (*p* < 0.0001) concentrations were significantly higher in individuals with PKU compared with controls** No significant difference in total homocysteine concentrations between individuals with PKU and controls (*p* = 0.059) Vitamin B6 concentrations for individuals with PKU were well above the respective lower range of normal recommended intake
Hvas et al. 2006 [49]	Europe (Denmark)	Cross-sectional study	Adults 18–43	• 31 individuals with PKU (mixed group of individuals with PKU on diet receiving LNAA and individuals not on diet or receiving LNAA) • Reference values	Nutritional outcomes	The majority of individuals with PKU had a vitamin B6 intake below the recommended daily intake. Eleven individuals (39%) received less than the recommended daily vitamin B12 intake from the diet. The authors concluded that adults with PKU were at an increased risk of developing vitamin B12 deficiency
Lage et al. 2010 [27]	Europe (Spain)	Cross-sectional study	Adults 19–42	• 22 individuals with PKU on a Phe-restricted diet • 42 healthy adult controls (fatty acid reference values) • Age- and sex-matched controls BMD used for Z-score calculation	Bone-related abnormalities	**DHA, EPA, and total n-3 fatty acids were significantly lower in individuals with PKU compared with controls (*p* < 0.05). The lack of essential n-3 fatty acid intake in the PKU diet might affect bone mineralization**
						Z-scores of BMD were lower than zero in individuals with PKU but not low enough to indicate presence of osteopenia (Z-score ≤ –2.5) or osteoporosis (–1 to –2.5)
Lubout et al. 2020 [28]	Europe (UK, France, The Netherlands, Poland, Spain)	Retrospective cohort study	Adults 19–53	• 183 individuals with PKU on a Phe-restricted diet (68% with cPKU, 22% with mild PKU, 10% with mild HPA) • Reference population BMD used for Z-score calculation	Bone-related abnormalities	**Significantly lower mean and median BMD Z-scores in individuals with PKU compared with the reference population (lumbar [*p* < 0.000]**^**e**^** femoral neck [*p* = 0.003]**^**e**^**, and total body BMD [*p* = 0.002]**^**e**^**)**^**f**^
						Mean BMD Z-scores were not significantly lower in individuals with PKU compared with the reference population at the radius level (*p* = 0.065)
Mazzola et al. 2016 [50]^c^	South America (Brazil)	Cross-sectional study	6–25	• 27 individuals with PKU on a Phe-restricted diet and L-AAM (13 cPKU and 14 mild PKU; 11 early diagnosed and 16 late-diagnosed) • 27 matched controls	Overweight/obesity	No significant differences between individuals with PKU and controls regarding body composition, including BMI, fat mass, fat-free mass, and ECM/BCM ratio^g^
Mezzomo et al. 2023 [51]	South America (Brazil)	Cross-sectional study	Adults 18–44	• 36 individuals with PKU (11 cPKU, 10 mild PKU, 15 unclassified PKU; 15 early diagnosed, 20 late diagnosed, one with no information on time of diagnosis) • 33 healthy controls	Overweight/obesity	No significant difference between PKU and control for bodyweight (*p* = 0.068), BMI (*p* = 0.612), abdominal circumference (*p* = 0.574), percentage body fat (*p* = 0.902), fat-free mass in kg (*p* = 0.902), and percentage fat-free mass (*p* = 0.344)
Millet et al. 2005 [36]	Europe (Spain)	Cross-sectional study	4–38	• 46 individuals with PKU on a Phe-restricted diet • Reference values from healthy controls	Bone-related abnormalities	**Significantly higher deoxypyridinoline in individuals with PKU than reference values (*p* = 0.031), suggesting higher bone resorption** **Ca/Cr was significantly higher in adults with PKU than reference values (*p* < 0.001), suggesting higher bone resorption** **Bone formation markers (osteocalcin [*p* < 0.028], BAP [*p* < 0.003]) were significantly decreased in individuals with PKU compared with reference values**
Modan-Moses et al. 2007 [29]^d^	Middle East (Israel)	Cross-sectional study	Adults 19–41	• 31 individuals with cPKU (adherent and non-adherent to a Phe-restricted diet; number of individuals in each group was unclear) • Age- and sex-matched controls used for Z-score calculation	Bone-related abnormalities	**Total body BMD Z-score (*p* = 0.002) and femoral neck BMD Z-score (*p* < 0.001) in individuals with cPKU were significantly lower than would be expected in the normal population** Osteopenia was detected in 11 individuals with cPKU (38.7%), while osteoporosis was detected in two individuals with PKU (6.5%). Decreased peak bone mass was demonstrated in 13 (42%) individuals with cPKU
						Lumbar BMD Z-score was lower than the normal population in individuals with cPKU but did not reach statistical significance^g^
Nagasaka et al. 2011 [37]	Asia (Japan)	Cross-sectional study	Adults 19–40	• 34 individuals with cPKU on a Phe-restricted diet • 36 healthy controls	Bone-related abnormalities	**Significantly higher bone resorption in individuals with cPKU than controls (*p* < 0.01)** **Significantly lower levels of osteoprotegerin (bone resorption inhibitor) in individuals with cPKU compared with controls (*p* < 0.001)**
						No significant difference in bone formation markers (BAP and osteocalcin) in individuals with cPKU compared with controls^g^
Ozel et al. 2013 [52]	Europe (seven countries + Turkey)	Cross-sectional study	Adults ≥ 19–NR for all centers	• 164 individuals with PKU on a Phe-restricted diet, with or without LNAA supplements and sapropterin dihydrochloride, or sapropterin dihydrochloride alone	Overweight/obesity	The majority of centers (five out of six, 83%) had less overweight adults with PKU than the respective general populations, but a higher rate of female obesity compared with males; in four centers the rate of female obesity was higher than the respective general population
Pérez-Dueñas et al. 2002 [30]	Europe (Spain)	Retrospective cohort study	Adults 19–33	• 14 individuals with PKU on a Phe-restricted diet • Reference population	Bone-related abnormalities	**Significantly lower levels of bone formation marker (BAP) in individuals with PKU than reference values** **(*p* < 0.0001)**
						Osteopenia was detected in 50% of individuals with PKU; 17.8% of individuals showed a Z-score < –2
						Bone formation marker (osteocalcin) was not significantly different between individuals with PKU and reference values^g^
Porta et al. 2008 [39]	Europe (Italy)	Case-controlled study	NR	• 20 individuals with PKU on a Phe-restricted diet • 20 healthy age-matched controls In vitro study using cells from individuals with PKU compared with cells from healthy controls	Bone-related abnormalities	**Individuals with PKU showed almost double the number of osteoclasts compared with controls (*p* = 0.001)**
Roato et al. 2010 [40]	Europe (Italy)	Cross-sectional study	6.5–22.7	• 40 individuals with PKU on a Phe-restricted diet • 40 healthy controls In vitro study using cells from individuals with PKU compared with cells from healthy controls	Bone-related abnormalities	**Osteoclastogenesis in individuals with PKU was significantly higher than in controls in unstimulated and stimulated cultures (*p* < 0.01)**
Robertson et al. 2013 [53]	Europe (UK)	Retrospective cohort study	Adults 16–41	• 236 individuals with PKU on a Phe-restricted diet • UK healthy population	Overweight/obesity	41% of individuals with PKU were within the healthy weight range 31% of individuals with PKU were within the overweight weight range 24% of individuals with PKU were within the obese weight range No statistically significant difference between the percentages of individuals with PKU in each BMI score category (overweight and obese) versus the general healthy population
Robinson et al. 2000 [54]^d^	Europe (UK)	Cross-sectional study	11–38	• 83 individuals with PKU (22 on a strict diet, 30 on a relaxed diet, 31 on an unrestricted diet) • Reference population for mean blood vitamin B12 concentration and erythrocyte folate concentration (normal population data for vitamin B12 obtained from the Office of Population Censuses and Surveys [The Dietary and Nutritional Survey of British Adults])	Nutritional outcomes	**Individuals with PKU on a relaxed or unrestricted diet had significantly lower vitamin B12 concentrations compared with the reference population (*p* = 0.0034 and *p* < 0.0001, respectively)**
						No significant difference was found in vitamin B12 concentration for those on a strict diet compared with the reference population^g^
						**Individuals with PKU on a strict, relaxed, or unrestricted diet had significantly higher erythrocyte folate concentration compared with the reference population (*p* < 0.0001, *p* < 0.0001, and *p* < 0.0001, respectively)**
Rocha et al. 2012 [55]	Europe (Portugal)	Cross-sectional study	3–47	• 89 individuals with PKU on a Phe-restricted diet • 79 healthy controls	Overweight/obesity	Individuals with PKU did not have a higher incidence of overweight and obesity (*p* = 0.293), body fat percentage (*p* = 0.581), or central obesity (*p* = 0.999) compared with controls
					Other	Individuals with PKU did not have a higher incidence of metabolic syndrome compared with controls (*p* = 0.365)
Rojas-Agurto et al. 2023 [33]^d^	South America (Chile)	Retrospective cohort study	Adults 19–27 PKU-1 19–26 Matched controls 20–27	• 10 individuals with PKU with a neonatal diagnosis of PKU on low-Phe nutritional treatment, including adequate supply of protein substitute, who kept strict follow-up • 10 matched controls	Musculoskeletal outcomes	**Left- and right-hand grip strength significantly decreased in individuals with PKU versus control (*p* = 0.04 and *p* = 0.01, respectively)**
					Bone-related abnormalities	No significant difference in spine or femoral BMD between individuals with PKU and their matched controls
					Overweight/obesity	No significant difference in bodyweight, BMI, WC, total or appendicular fat mass, or total or appendicular fat-free mass between individuals with PKU and their matched controls
					Nutritional outcomes	**Significantly higher serum folic acid in individuals with PKU versus matched controls (*p* < 0.01)** Levels of vitamin B12 and D3 were higher in individuals with PKU versus matched controls (B12 within reference range in both groups, D3 below reference for control group and within reference for PKU group) but differences between groups were not statistically significant^g^ Homocysteine levels were lower in individuals with PKU versus matched controls but levels in both groups were within reference range and the difference between groups was not statistically significant^g^ **Intake of vitamin B12, folic acid, vitamin D, and calcium were significantly higher in individuals with PKU versus matched controls (*p* < 0.01 for vitamin B12, vitamin D, and folic acid, *p* = 0.04 for calcium)**
Schoen et al. 2022 [56]	North America (USA)	Cross-sectional study	Adults 18–70 Individuals with PKU on diet therapy 18–50 NHANES dataset 18–70	• 17 individuals with PKU on diet therapy • 7267 reference sample from NHANES dataset	Overweight/obesity	Overall, overweight/obesity was less common in individuals with PKU on diet therapy than in controls (64.7% versus 71.5%), driven by a much lower percentage of obese individuals (23.5% versus 41.5%); however, being overweight was more common in individuals with PKU on diet therapy than in controls (41.2% versus 30.0%)^h^
					Nutritional outcomes	Adults with PKU on diet therapy were less likely to achieve adequate intake of choline than controls (5.6% versus 9.5%) There was a very low probability of inadequate intake of nutrients affecting choline metabolism (vitamin B6, vitamin B12, folate, methionine) in both groups
Schwahn 1998 [42]	Europe (Germany)	Cross-sectional study	5–28	• 14 individuals with PKU on a Phe-restricted diet • 14 healthy controls	Bone-related abnormalities	**Significant reduction of SBMD in all individuals with PKU compared with controls (*p* < 0.01)**
						TBMD in individuals with PKU was slightly decreased in adolescents and adults compared with controls but did not reach statistical significance^g^
Stroup et al. 2017 [38]	North America (USA)	Crossover study	Adults 16–35	• Eight individuals with PKU on a Phe-restricted diet (four with cPKU, four with a variant form of PKU) • Reference values	Bone-related abnormalities	Average bone-specific alkaline phosphatase concentrations were above the reference range for male individuals with PKU only
Sumanszki et al. 2019 [57]^d^	Europe (Hungary)	Cross-sectional study	Adults 18–49	• 69 individuals with PKU on a Phe-restricted diet and/or receiving AAM. Individuals were grouped based on their adherence to a low-protein diet (30 in low-adherence group and 37 in good-adherence group) and AAM consumption (25 in reduced AAM-intake group and 42 in adequate AAM-intake group) • 50 healthy controls	Other	**Thyroid function: TSH level was significantly lower (*p* = 0.002) and free T**_**4**_** was significantly higher** **(*p* = 0.022) in the individuals with PKU compared with controls** No signs of thyroiditis were observed in any of the individuals with PKU No significant differences were found in thyroid volume (*p* = 0.462) and in thyroid nodule incidence (*p* = 0.174) between individuals with PKU and controls overall
Trefz et al. 2019 [15]	Europe (Germany)	Retrospective cohort study	Adults 18–96	• 377 individuals with PKU; some individuals received sapropterin dihydrochloride (n < 5), some individuals were on a diet (*n* = 52), for some individuals it was not clear whether they were on a diet • 3770 matched healthy controls	Hypertension	More than a third of individuals with PKU suffered from essential (primary) hypertension but prevalence ratio was not significantly different compared with controls^i^
					Overweight/obesity	**Prevalence ratio of overweight and obesity in individuals with PKU was significantly higher compared with controls (95% CI did not include a value of 1.0)**^i^
					Cardiovascular outcomes	**Prevalence ratio of chronic ischemic heart disease in individuals with PKU was significantly higher compared with controls (95% CI did not include a value of 1.0)**^i^
					Diabetes	**Prevalence ratio of unspecified diabetes mellitus in individuals with PKU was significantly higher compared with controls (95% CI did not include a value of 1.0)**^i^
						Prevalence ratio of type 2 diabetes mellitus in individuals with PKU was not significantly higher compared with controls^i^
					Bone-related abnormalities	Prevalence ratio of osteoarthritis of the knee and spondylosis in individuals with PKU was not significantly different compared with controls^i^
					Gastrointestinal disorders	**Prevalence ratio of infectious gastroenteritis and colitis in individuals with PKU was significantly higher compared with controls (95% CI did not include a value of 1.0)**^i^
						Prevalence ratio of gastritis and duodenitis in individuals with PKU was not significantly higher compared with controls^i^
					COPD/asthma	**Prevalence ratio of asthma in individuals with PKU was significantly higher compared with controls** **(95% CI did not include a value of 1.0)**^i^
					Dermatologic disorders	**Prevalence ratio of “other and unspecified dermatitis” in individuals with PKU was significantly higher compared with controls (95% CI did not include a value of 1.0)**^i^
						Prevalence ratio of melanocytic nevi in individuals with PKU was not significantly higher compared with controls^i^
					Abdominal and pelvic pain	Prevalence ratio of abdominal and pelvic pain in individuals with PKU was not significantly higher compared with controls^i^
					Other	**Prevalence ratio of some other comorbidities in individuals with PKU was significantly higher compared with controls (95% CI did not include a value of 1.0): dorsalgia; other and unspecified dorsopathies; other disorders of the urinary system; menopausal and other perimenopausal disorders; dizziness and giddiness; other and unspecified soft tissue disorders; and adverse events, not elsewhere classified**^i^
						Prevalence ratio of some other comorbidities in individuals with PKU was not significantly higher compared with controls: refraction and accommodation disorders, other non-inflammatory disorders of the vagina, acute upper respiratory infections of multiple and unspecified sites, other non-toxic goiter, other hypothyroidism, vasomotor and allergic rhinitis, congenital deformities of feet, and varicose veins of lower extremities^i^
Vugteveen et al. 2011 [58]	Europe (The Netherlands)	Cross-sectional study	1–37	• 75 individuals with PKU on a Phe-restricted diet • Reference values	Nutritional outcomes	18 individuals with PKU had abnormal concentrations of vitamin B12, MMA, and/or homocysteine compared with reference values MMA concentration (*p* = 0.000), plasma homocysteine concentration (*p* = 0.000), and metabolic control (*p* = 0.002) were negatively related to serum vitamin B12 concentrations
Weigel et al. 2008 [59]	Europe (Germany)	Cross-sectional study	0.2–39	• 30 individuals with cPKU on a Phe-restricted diet • 50 healthy controls	Nutritional outcomes	**Individuals with PKU had significantly lower free carnitine concentrations compared with controls (*p* < 0.001)**
Zeman et al. 1999 [31]	Europe (Czech Republic)	Cross-sectional study	6–29	• 44 individuals with cPKU on a Phe-restricted diet • Reference values used for Z-score calculation	Bone-related abnormalities	Significant positive correlation between total and lumbar spine BMD in individuals with PKU (*p* < 0.001)
						Lumbar spine BMD (Z-score < –1) was decreased in 20 individuals with PKU and total body BMD (Z-score < –1) was decreased in 14 individuals with PKU
						Normal BMD was found in 24 (54%) individuals with PKU

Results in bold font showed a statistically significant difference between groups

*Abbreviations: AAM* amino acid mixture, *AV* assigned value, *BAP* bone-specific alkaline phosphatase, *BH4* tetrahydrobiopterin, *BMD* bone mineral density, *BMI* body mass index, *Ca/Cr* calcium/creatinine index, *CCI* Charlson Comorbidity Index, *CI* confidence interval, *CIMT* carotid intima media thickness, *COPD* chronic obstructive pulmonary disease, *cPKU* classical PKU, *DHA* docosahexaenoic acid, *ECM/BCM* extracellular mass/body cell mass, *EPA* eicosapentaenoic acid, *GERD* gastroesophageal reflux disease, *HOMA-IR* homeostasis model assessment insulin resistance, *HPA* hyperphenylalaninemia, *L-AAM* L-amino acid mixture, *LNAA* large neutral amino acids, *MMA*, methylmalonic acid, *NHANES* National Health and Nutrition Examination Survey, *NR* not reported, *Phe* phenylalanine, *PKU* phenylketonuria, *PS* protein substitute, *SBMD* spongy bone mineral density, *SD* standard deviation, *SIRS* systemic inflammatory response syndrome, *SSI* strength strain index, *T*_*4*_ thyroxine, *TMBD* total bone mineral density, *TSH* thyroid-stimulating hormone

^a^Number of individuals ≥ 16 years with a documented medical resource use

^b^Study appears in Tables 2 and 5

^c^Study appears in Tables 2, 3, and 5

^d^Study appears in both Tables 2 and 3

^e^Significantly different from the general population (*p* < 0.05) after post-hoc adjustment of the *p* value by the Bonferroni method (multiplying *p* value by the number of tests)

^f^Although the mean BMD in early treated individuals with PKU was significantly lower compared with the general population, BMD was within the normal range in most individuals

^g^Authors conducted statistical testing and reported that the difference between groups was not significant but did not report the *p* value

^h^Data taken from Table 1 within the publication

^i^Differences between groups were tested using 95% CI of prevalence ratio values

**Fig. 3:**
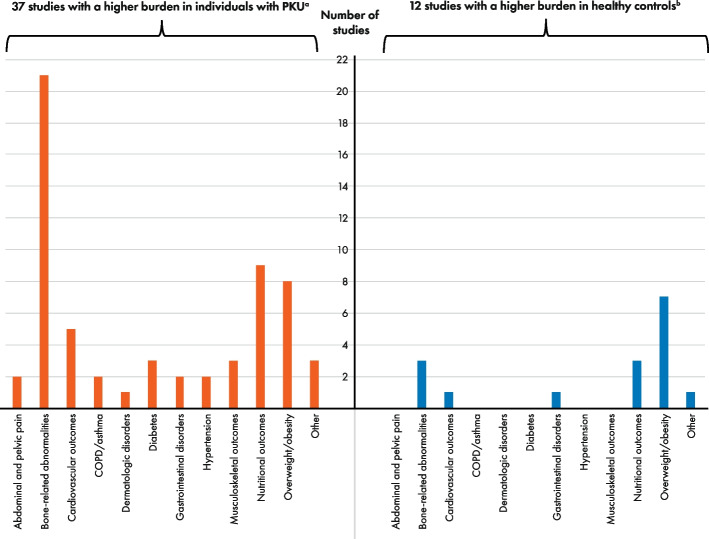
Burden of somatic comorbidities in individuals with PKU on a Phe-restricted diet versus healthy controls or reference values as assessed by vote counting. Abbreviations: COPD, chronic obstructive pulmonary disease; Phe, phenylalanine; PKU, phenylketonuria. Note: Total number of studies = 38. ^a^Studies with a higher burden of ≥ 1 comorbidity or outcome measure, for a given comorbidity category, in individuals with PKU who adhered to a Phe-restricted diet. ^b^ Studies with a higher burden of ≥ 1 comorbidity or outcome measure, for a given comorbidity category, in healthy control individuals or a normal reference population. Some studies reported more than one comorbidity or outcome measure per category. Studies reporting a differing direction of effect between comorbidities or outcome measures within a category, are indicated below. Details of studies with consistent direction of effect are not listed below (but are included in Table 2). Vote counting was conducted on the basis of numerical differences in the direction of effect, regardless of statistical significance or clinical relevance. **Abdominal and pelvic pain:** Higher burden in PKU (*n* = 2) [15, 22]. **Bone-related abnormalities:** Higher burden in PKU (*n* = 21) [15, 22, 24–42]; negative BMD for distal radius, total body, and trabecular bone; proximal radius, total body, and worse measures of bone geometry and strength in PKU group [24], lumbar and femoral BMD Z-score < –2 in 5.0% and 7.0% of all patients, negative median BMD in adults for hip bone, higher percentage of all patients with fracture history in PKU group [26], lower vitamin D status, higher concentrations of all bone resorption markers, lower concentrations of all bone formation markers except alkaline phosphatase, and higher calcium and phosphorus excretion in PKU group [37]; higher burden in controls (*n* = 3) [24, 26, 37], positive BMD for proximal radius cortical bone in PKU group [24], positive median BMD in adults for femur in PKU group [26], higher concentration of alkaline phosphatase in PKU group [24, 37]. **Cardiovascular outcomes:** Higher burden in PKU (*n* = 5) [15, 22, 43, 46, 47], higher arterial stiffness in PKU group [47]; higher burden in controls (*n* = 1), higher intima media thickness in control group [47]. **COPD/asthma:** Higher burden in PKU (*n* = 2) [15, 22]. **Dermatologic disorders:** Higher burden in PKU (*n* = 1) [15]. **Diabetes:** Higher burden in PKU (*n* = 3) [15, 22, 44]. **Gastrointestinal disorders:** Higher burden in PKU (*n* = 2) [15, 22], numerically higher frequency of diverticular disease of intestine in individuals with PKU versus non-PKU control [22]; higher burden in controls (*n* = 1) [22], numerically higher frequency of gastritis and duodenitis in non-PKU controls versus individuals with PKU and numerically higher frequency of constipation in non-PKU controls compared with the early-diagnosed PKU subgroup only (null effect on constipation between the overall PKU group and non-PKU controls) [22]. **Hypertension:** Higher burden in PKU (n = 2) [15, 22]. **Musculoskeletal outcomes:** Higher burden in PKU (*n* = 3) [22, 24, 33], muscle size and performance were preserved in individuals with PKU and regression lines were comparable to the reference population (null effect, excluded from vote counting [24]). **Nutritional outcomes:** Higher burden in PKU (*n* = 9) [22, 26, 45, 48, 49, 54, 56, 58, 59], decreased concentration of vitamin B12 in relaxed diet and unrestricted diet groups versus control [54], concentration of vitamin D, selenium and zinc below reference range [26], individuals with PKU were less likely to achieve adequate choline intake compared with controls [56]; higher burden in controls (*n* = 3) [26, 33, 54], increased concentration of vitamin B12 in strict diet group and increased concentration of folate in all diet groups versus control (within or above normal range) [54], concentration of magnesium, folate, vitamin B12 and B6 above reference range [26, 54], analysis only considered the PKU population who consumed adequate protein substitute without Phe and maintained strict metabolic follow-up [33]; one study investigating mean probability of adequacy for vitamin B6, B12, and folate reported a null effect for individuals with PKU on Phe-restricted diet with medical food and dietary supplements versus healthy controls (excluded from vote counting) [56]. **Overweight/obesity:** Higher burden in PKU (*n* = 8) [15, 22, 43, 44, 50–53], percentage of females with BMI > 30 kg/m2 was higher than in all UK countries assessed, percentage of females with BMI > 25 kg/m2 was higher than in Northern Ireland only [53], percentages of individuals with PKU who were obese was higher than in the general population in 2/6 centers [52], fat-free mass (Kg) was numerically lower in individuals with PKU versus healthy control [51], no controls below normal range for BMI as opposed to PKU group; higher burden in controls (*n* = 7) [33, 50–53, 55, 56], adults > 16 years subgroup had higher prevalence of overweight/obesity in control versus PKU [55] but percentage body fat was equal (excluded from vote counting) [55], percentage of males with BMI > 25 and > 30 kg/m2 was higher than in all UK countries assessed, percentage of females with BMI > 30 kg/m2 was higher than in England and Scotland only [53], percentages of individuals with PKU who were overweight and obese were lower than those in the general population in 5/6 and 3/6 study centers, respectively (percentage of individuals with PKU who were obese was the same as that for the general population in 1/6 centers (excluded from vote counting) [52], bodyweight and BMI was numerically lower in individuals with PKU versus healthy control but both groups were only borderline overweight, percentage fat-free mass was numerically higher in individuals with PKU versus healthy control [51], bodyweight, percentage fat mass, and BMI were numerically less in individuals with PKU than healthy controls and BMI of more controls was above normal range, percentage fat-free mass was higher in individuals with PKU than in healthy controls [50]. **Other:** Higher burden in PKU (*n* = 3) [15, 22, 57]; higher burden in controls (*n* = 1) [55]. In the majority of studies, all individuals were on a Phe-restricted diet, with the following exceptions: one study (*n* = 83) of which 31 were on an unrestricted diet – no formal protein restriction and not taking amino acid supplements, 30 were on a relaxed diet – total protein intake of approximately 1 g/kg/d (50% from natural protein/ 50% from amino acid, vitamin and mineral supplements), and 22 were on a strict low-Phe diet, including amino acid, vitamin, and mineral supplements [54]; one study with a mixture of individuals on and not on a Phe-restricted diet [25]; one study in which some individuals were on sapropterin dihydrochloride or pegvaliase in addition to a Phe-restricted diet [25]; one study in which some individuals received sapropterin dihydrochloride, some were on a Phe-restricted diet, and, for some, it was not clear whether they were on a Phe-restricted diet or not [15]; one study in which some individuals were treated with sapropterin dihydrochloride in addition to dietary treatment [46]; one study (*n* = 164) in which the majority of individuals were on a Phe-restricted diet, up to 20 adults received additional BH4, and up to 11 adults received BH4 alone [52]; one study (*n* = 1911) in which 29% of individuals received amino acid supplementation and 5% received sapropterin dihydrochloride (unclear if remaining individuals were on Phe-restricted diet) [22]; one study in which 80% of individuals were on Phe-restricted diet and 20% were on sapropterin dihydrochloride with or without amino acid supplementation [32]; one study in which individuals on BH4 were excluded and it was unclear whether individuals were on a Phe-restricted diet or not [51]

A higher burden of ≥ 1 comorbidity (or outcome measure) in individuals with PKU on a Phe-restricted diet with or without pharmacologic therapy was indicated by 37 of 38 studies included in the vote-counting analysis, versus a higher burden of ≥ 1 comorbidity (or outcome measure) in healthy controls or reference values in 12 studies (Fig. 3). The most commonly reported somatic comorbidities with a higher burden in those on a Phe-restricted diet with or without pharmacologic therapy were bone-related abnormalities (*n* = 21), nutritional outcomes (*n* = 9), overweight/obesity (*n* = 8), and cardiovascular outcomes (*n* = 5). The most commonly reported somatic comorbidities with a higher burden in healthy controls or reference values were overweight/obesity (*n* = 7), bone-related abnormalities (*n* = 3), and nutritional outcomes (*n* = 3).

**Fig. 4:**
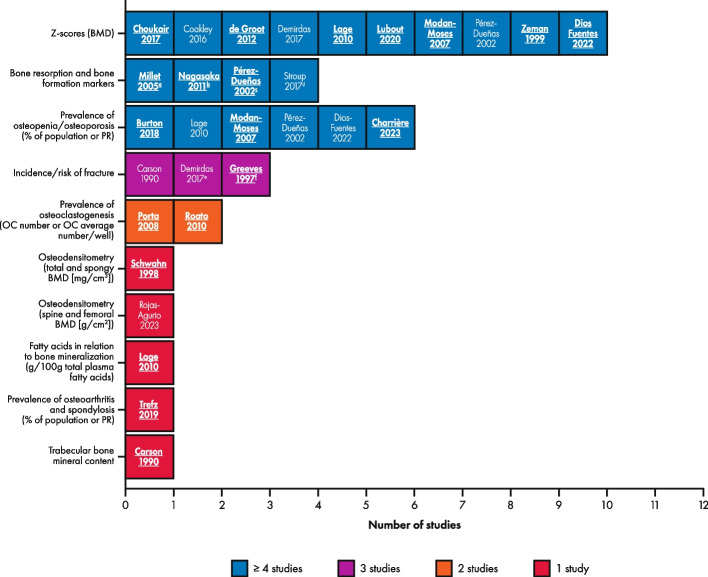
Overview of measures used to report bone-related abnormalities in individuals with PKU on a Phe-restricted diet versus healthy controls or reference values. Abbreviations: BMD, bone mineral density; OC, osteoclastogenesis; Phe, phenylalanine; PKU, phenylketonuria; PR, prevalence ratio. Studies in bold font showed a statistically significant difference between groups. All 21 studies indicated a higher clinical burden of ≥ 1 outcome measure in the PKU group (or a particular subgroup) compared with healthy controls; with 15 studies reporting a statistically significant difference [16, 22, 24, 27–30, 34–37, 39–42], two studies that did not find a statistically significant difference for any outcome measure [15, 33], and four studies that did not test for statistical significance between PKU group and controls [25, 26, 31, 32]; in seven studies the difference between groups was not statistically significant for all outcome measures [29], outcomes [24, 27, 28, 30, 37], or in the comparison of the overall PKU population [35]. ^a^Units: osteocalcin (μg/L), bone alkaline phosphatase (BAP; μg/L), deoxypyridinoline (μmol/mol creatine), calcium/creatine index (no units reported); ^b^ Units: osteocalcin (ng/mL), BAP (U/I), intact parathyroid hormone (pg/mL), 1,25 (OH)_2_ vitamin D (pg/mL), 25 (OH) vitamin D (ng/mL), urinary deoxypyridinoline (nmol/mmol creatinine), urinary N-telopeptides of type collagen (nmol/mmol creatinine), ICTP (pyridinoline cross-linked telopeptide domain of type I collagen; ng/mL), osteoprotegerin (pmol/L), urinary calcium/creatine index (mmol/mmol creatinine), urinary phosphorus/creatine index (mmol/mmol creatinine); ^c^ Units: osteocalcin (no reported units), BAP (μg/L); ^d^ Units: BAP (μg/L); ^e^ Lifetime fracture prevalence was measured as percentage of the population; ^f^ Risk of fracture was measured between 0 and 20 years of age using a Kaplan–Meier graph (cumulative proportion with fracture vs age)

Bone-related abnormalities encompass a range of features; abnormalities reported in individuals with PKU included reduced BMD, measured by Z-scores [24–32] or g/cm^2^ [33]; presence of osteopenia/osteoporosis [16, 22, 27, 29, 30]; lower distal radius [24]; reduced cortical thickness and strength-strain index [24]; greater risk of fracture [26, 34, 35]; reduced levels of bone formation markers and/or increased levels of bone resorption markers [30, 36–38]; higher prevalence of osteoarthritis of the knee [15]; higher prevalence of spondylosis [15]; and increased presence of osteoclastogenesis [39, 40]. Bone-related abnormalities reported in healthy controls included reduced BMD [24, 26]; reduced cortical density [24]; and high levels of bone formation markers [37]. The most reported outcome measures for bone-related abnormalities (reported in ≥ 4 studies) were Z-scores (*n* = 10), markers for bone resorption and bone formation (*n* = 4), and prevalence of osteopenia/osteoporosis (*n* = 6) (Fig. 4). There was no numerical difference between groups in femoral BMD in one study [33]; therefore, this was not captured in the vote counting. Statistical significance of the difference in bone-related abnormalities between those on a Phe-restricted diet with or without pharmacologic therapy versus healthy controls or reference values was assessed in 17 [15, 16, 22, 24, 27–30, 33–37, 39–42] of the 22 studies (including one study [16] not included in the vote counting), of which the majority showed a higher burden of ≥ 1 outcome measure in the PKU group; results are reported in Fig. 4 and Table 2.

Of ten studies reporting nutritional outcomes included in the vote-counting analysis shown in Fig. 3, nine studies reported a higher burden of ≥ 1 outcome measure in those on a Phe-restricted diet compared with healthy controls or reference values [22, 26, 45, 48, 49, 54, 56, 58, 59] whereas three studies reported a higher burden of ≥ 1 outcome measure in healthy controls or reference values [26, 33, 54]; results are reported in Table 2. This included significantly lower free carnitine concentrations [59]; significantly higher percentages of individuals with vitamin D deficiency and iron deficiency anemia [22] abnormal concentrations of vitamin B12, methylmalonic acid, and homocysteine [58]; higher concentrations of folate [33, 48, 54], cobalamin, and homocysteine [48]; concentrations of magnesium, folate, vitamin B12, and vitamin B6 above or within the reference range [26]; higher concentrations of vitamin B12 and vitamin D3 and lower concentrations of homocysteine that were within reference range (but the differences between groups were not statistically significant) [33]; lower concentrations of vitamin B12 [45, 49, 54], vitamin B6 [49], selenium, pre-albumin, folate, vitamin D, ferritin and zinc (although the difference versus the normal reference was not statistically significant [45]); concentrations of vitamin D and selenium within or below the reference range [26]; and a lower likelihood of achieving adequate choline intake but a very low probability of achieving inadequate intake of vitamin B6, vitamin B12, folate, and methionine in both groups [56]. One study [45] observed significant correlations between changes in nutritional outcomes and participant age (≤ 18 years versus > 18 years): total protein and pre-albumin levels increased with age (*p* = 0.002 and *p* < 0.0001, respectively), whereas calcium and phosphorus decreased with age (*p* = 0.015 and *p* < 0.0001, respectively). In the same study, vitamin B12 levels were significantly lower in BH4-treated versus BH4-untreated participants [45].

Twelve studies reported outcome measures relating to overweight/obesity [15, 16, 22, 33, 43, 44, 50–53, 55]; results are reported in Table 2 and Fig. 3 (one study was excluded from the vote-counting analysis [16]). Four studies [15, 16, 22, 43] reported a significantly higher body mass index (BMI), prevalence ratio, or percentage of individuals who were overweight and/or obese in those with PKU versus healthy controls. The statistical significance of the difference between groups was maintained for a subgroup of early diagnosed individuals in one of these studies [22]; one study [44] reported numerically higher proportions of overweight/obesity among the PKU population versus healthy controls (39% versus 25%) but the statistical significance of the difference was not reported; in one study [52] the rate of obesity in females with PKU was higher than in the respective general (non-PKU) population in four of six centers, but the overall rate of overweight participants was lower in five of six centers studied; and being overweight was more common and obesity was less common in individuals with PKU compared with the reference dataset in one study [56]. However, two studies [53, 55] reported no significant difference in the proportions of the PKU population who were overweight/obese compared with the control population and two studies reported no significant difference in bodyweight, BMI, total fat mass, total fat-free mass [33, 50], appendicular fat-free mass, appendicular fat-free mass index, and waist circumference (WC) [33] between the PKU population on a Phe-restricted diet and heathy controls. Three studies did not assess statistical significance of the differences between populations: in the first study [52], the proportion of obese females with PKU was higher than in the respective general (non-PKU) population in four of six centers studied, but numerically lower proportions of obese individuals were reported overall in three of six centers, higher proportions were reported in two of six centers, and the same proportion was reported in one of six centers in the PKU group versus the control group, while a lower proportion of overweight/obese individuals in the PKU group versus the control group was observed in five of six centers; the second study [51] reported numerically lower WC and BMI and a numerically higher percentage of fat-free mass in individuals with PKU versus controls, but numerically lower absolute fat-fee mass (kg) in those with PKU versus controls; and the third study [56] reported numerically higher percentages of overweight individuals and numerically lower percentages of obese individuals in the PKU group versus controls.

Five studies reported cardiovascular outcome measures [15, 22, 43, 46, 47], including an increase in arterial stiffness markers (*n* = 2) [46, 47], increased prevalence of ischemic heart disease (*n* = 2) [15, 22], higher heart rate and blood pressure (*n* = 1) [43], and increase in carotid intima media thickness (*n* = 1) [47]. Statistical significance of the difference in cardiovascular outcomes between those on a Phe-restricted diet with or without pharmacologic therapy versus healthy controls or reference values was assessed in four [15, 22, 43, 46] out of five [15, 22, 43, 46, 47] studies and included significantly higher resting heart rate and systolic blood pressure [43], markers of arterial stiffness [46], and prevalence of chronic ischemic heart disease [15, 22] in individuals with PKU versus healthy controls; results are reported in Table 2 and Fig. 3.

#### Individuals with PKU on pharmacologic therapy with or without a Phe-restricted diet versus those on Phe-restricted diet alone

One study [56] investigated choline nutriture in adults and children with PKU receiving pegvaliase (*n* = 33 adults), sapropterin dihydrochloride (*n* = 21 adults), or dietary therapy alone (*n* = 17 adults). This study found that adults receiving pegvaliase were most likely to exceed adequate intake of choline (14.82%, standard error [SE] 4.48), while adults on dietary therapy alone were least likely (5.59%, SE 2.95). In general, however, there was a very low probability of inadequate intake of nutrients affecting choline metabolism (vitamin B6, vitamin B12, folate, and methionine) among adults with PKU. In this study [56], the pegvaliase group had the highest percentage of overweight/obesity compared with those on dietary therapy alone and those on sapropterin dihydrochloride (81.8%, 64.7%, and 61.9%, respectively), driven by a higher rate of obesity than in individuals on either sapropterin dihydrochloride or dietary therapy alone (48.5%, 38.1%, and 23.5%, respectively); however, the percentage of individuals who were overweight was highest in the group on dietary therapy alone (41.2% versus 33.3% and 23.8% for those on pegvaliase and sapropterin dihydrochloride, respectively).

#### Individuals with PKU adherent to a Phe-restricted diet versus a non-adherent population

**Fig. 5:**
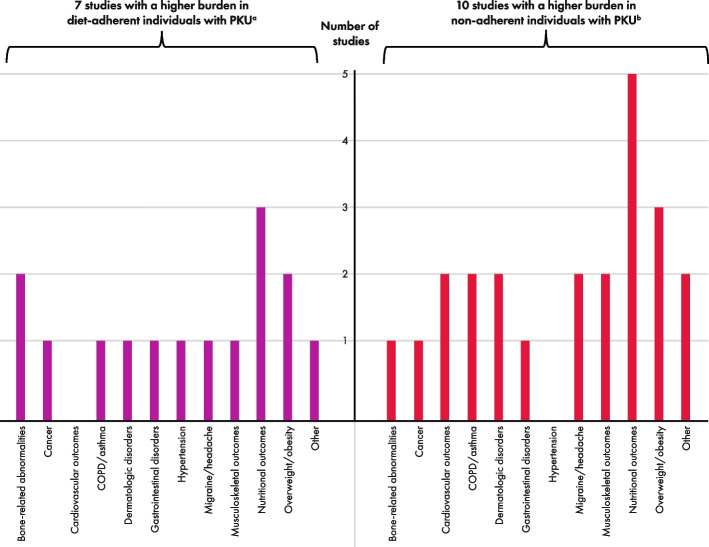
Burden of somatic comorbidities in individuals with PKU adherent to versus those not adherent to a Phe-restricted diet as assessed by vote counting. Abbreviations: COPD, chronic obstructive pulmonary disease; Phe, phenylalanine; PKU, phenylketonuria. Note: Total number of studies = 11. ^a^Studies with a higher burden of ≥ 1 comorbidity or outcome measure, for a given comorbidity category, in individuals with PKU who adhered to a Phe-restricted diet. ^b^ Studies with a higher burden of ≥ 1 comorbidity or outcome measure, for a given comorbidity category, in individuals with PKU who did not adhere to a Phe-restricted diet. Studies reporting more than one comorbidity or outcome measure per category, or those with a differing direction of effect between comorbidities or outcome measures within a category, are indicated below. Vote counting was conducted on the basis of numerical differences in the direction of effect, regardless of statistical significance or clinical relevance. **Bone-related abnormalities:** higher burden in adherent (*n* = 2) [29, 33], lower lumbar, femoral neck, and total body BMD Z-scores [29], lower spine BMD and null effect for femoral BMD (excluded from vote counting) [33]; higher burden in non-adherent (*n* = 1) [63]. **Cancer:** higher burden in adherent (*n* = 1), higher incidence in discontinued and restarted (group 3) compared with never treated (group 4) and off-diet (group 2) [60]; higher burden in non-adherent (*n* = 1), higher incidence in off diet (group 2) compared with adherent since birth (group 1) [60]; no reports of cancer in either group (excluded from vote counting) [61]. **Cardiovascular outcomes:** higher burden in non-adherent (*n* = 2) [60, 61], heart disease in larger proportion of participants [61], higher incidence of cardiovascular symptoms in off-diet (group 2) compared with discontinued and restarted (group 3) and adhered since birth (group 1), and higher incidence in never treated (group 4) compared with all other groups [60]. **COPD/asthma:** higher burden in adherent (n = 1), higher incidence of asthma in discontinued and restarted (group 3) than in off-diet (group 2) and never treated (group 4) [60]; higher burden in non-adherent (*n* = 2) [60, 61], higher incidence of asthma in never treated (group 4) than in adhered since birth (group 1) [60], asthma reported in larger proportion of participants [61]. **Dermatologic disorders:** higher burden in adherent (*n* = 1), higher incidence of dermatologic symptoms in discontinued and restarted (group 3) than in off-diet (group 2) and never treated (group 4) [60]; higher burden in non-adherent (*n* = 2) [60, 61], eczema reported in larger proportion of participants [61], higher incidence of dermatologic disorders in off diet (group 2) compared with adherent since birth (group 1), higher incidence of dermatologic disorders in never treated (group 4) compared with adherent since birth (group 1) and off-diet (group 2) [60]. **Gastrointestinal disorders:** higher burden in adherent (*n* = 1), higher incidence in discontinued and restarted (group 3) compared with off diet (group 2) and never treated (group 4) [60]; higher burden in non-adherent (*n* = 1), higher incidence in discontinued and restarted (group 3) compared with adherent since birth (group 1) [60]. **Hypertension:** higher burden in adherent (*n* = 1), hypertension reported in larger proportion of participants [61]. **Migraine/headache:** higher burden in adherent (*n* = 1), higher incidence of headaches in adherent since birth (group 1) compared with off-diet (group 2) and higher incidence of headaches in discontinued and restarted (group 3) compared with off-diet (group 2) and never treated (group 4) [60]; higher burden in non-adherent (*n* = 2) [60, 61], higher incidence of headaches in discontinued and restarted (group 3) compared with adherent since birth (group 1) [60], headaches reported in larger proportion of participants [61]. **Musculoskeletal outcomes:** higher burden in adherent (*n* = 1), decreased left and right hand-grip strength in adherent versus non-adherent [33]; higher burden in non-adherent (*n* = 2) [60, 63], higher incidence of arthritis/musculoskeletal symptoms in discontinued and restarted (group 3) compared with adherent since birth (group 1) [60]. **Nutritional outcomes:** higher burden in adherent (*n* = 3) [32, 45, 62], decreased concentrations of total protein and pre-albumin [45], lower concentrations of vitamin B12 and niacin in males [62], lower vitamin B12 in controlled versus uncontrolled population and almost significant increase in percentage of individuals with vitamin B12 deficiency [32]; higher burden in non-adherent (*n* = 5) [5, 33, 45, 54, 62], lower concentrations of phosphorus and vitamin B12 [45], lower concentrations of vitamin B12 and niacin in females as well as all other nutrients measured [62], lower intakes of iron, zinc, vitamin D3, magnesium, calcium, selenium, iodine, vitamin C, vitamin A, and copper, which were below UK Reference, and lower intakes of thiamin, riboflavin, niacin, vitamin B6, and phosphorus, which met UK Reference [5], lower concentrations of vitamin B12 and folate but levels were within or above normal range [5, 45, 54, 62], significantly lower serum vitamin D3 and vitamin B12, below reference range, versus above reference range in adherent, lower serum folic acid and higher serum homocysteine but both within reference range [33]. **Overweight/obesity:** higher burden in adherent (*n* = 2) [33, 62], obesity reported in larger proportion of participants [61], increased total fat free mass in non-adherent versus adherent [33]; higher burden in non-adherent (*n* = 3) [32, 33, 63], increased total fat mass, bodyweight, WC and BMI in non-adherent versus adherent (BMI within normal range for adherent), and decreased appendicular fat free mass in non-adherent versus adherent [33], significantly increased BMI in uncontrolled vs controlled (total population and women only), numerical increase in BMI of uncontrolled vs controlled men (controlled groups within normal range) [32]. **Other:** higher burden in adherent (*n* = 1) [60], higher incidence of otolaryngologic symptoms in adherent since birth (group 1) compared with off diet (group 2) and never treated (group 4), and gynecologic symptoms in adherent since birth (group 1) compared with never treated (group 4), higher incidence of arthritis/musculoskeletal symptoms in discontinued and restarted (group 3) compared with never treated (group 4) and off-diet (group 2), higher incidence of ophthalmologic and gynecologic symptoms in adherent since birth (group 1) compared with never treated (group 4) [60]; higher burden in non-adherent (*n* = 2) [57, 60], higher incidence of gynecologic and ophthalmologic symptoms in off diet (group 2) compared with adherent since birth (group 1), higher incidence of otolaryngologic symptoms in discontinued and restarted (group 3) compared with adherent since birth (group 1), higher incidence of ophthalmologic symptoms in off diet (group 2) compared with discontinued and restarted (group 3), higher incidence of gynecologic symptoms in discontinued and restarted (group 3) compared with never treated (group 4), and higher incidence of ophthalmologic symptoms in never treated (group 4) compared with adherent since birth (group 1) [60], poorer thyroid function as measured by serum TSH, UIC and UIC/Cr [57]*. ***Definitions of adherence versus non-adherence:** Adamczyk et al. 2011. [63], all individuals on Phe-restricted diet from within the first month of life, with blood Phe level assessment at least every second month: subgroup 2a (adherent) had recommended blood Phe levels for treated patients (2–10 mg/dL for children > 12 years), subgroup 2b (non-adherent) had blood Phe levels above the recommended level; Crujeiras et al. [45], those with high adherence to a natural protein restricted diet and supplementation with Phe-free amino acids mixture versus those with low adherence; Dios-Fuentes et al. [32], good metabolic control was defined as Phe levels < 600 µmol/L; Green et al. [5], minimum of 20 g protein equivalent from a low-Phe protein substitute per day for ≥ 1 month prior to inclusion with good adherence versus maximum of 20 g protein equivalent from a low-Phe protein substitute per day for ≥ 1 month prior to inclusion and blood Phe ≥ 600 µmol/L (of *n* = 14 in this group: *n* = 2 with 20 g of protein equivalent and no natural protein restriction; *n* = 1 with low protein diet but no low-Phe protein substitute; *n* = 11 with unrestricted diet and no low-Phe protein substitute); Guest et al. [60], remaining on Phe-restricted diet since < 1 year of age (group 1) versus discontinuation by 15–25 years of age (group 2) versus those off diet by 15–25 years of age but restarted diet at a mean of 30 years of age (group 3) versus those never treated (group 4); Koch et al. [61], Phe-restricted diet from infancy until ≥ 10 years of age and taking medical food as the primary protein source versus discontinuation of dietary restriction by age 10; Moden-Moses et al. [29], classified as diet-adherent or non-adherent based on self-report; Rojas-Agurto et al. [33], participants with a neonatal diagnosis of PKU, who continued with nutritional treatment, received an adequate supply of protein substitute without Phe, and kept strict follow-up were categorized as adherent, participants with a neonatal diagnosis of PKU, who discontinued the protein substitute and micronutrient supplementation (calcium, iron, and zinc) at 18 years of age and stopped attending metabolic control appointments; Robinson et al. [54], strict low-Phe diet with amino acid, mineral, and vitamin supplements versus no formal protein restriction and no amino acid vitamin and mineral supplementation (those on a total protein intake of approximately 1 g/kg/d with roughly 50% of this from natural protein and 50% from amino acid, mineral, and vitamin supplements were not included in the vote counting); Schulz et al. [62] taking amino acid mixture versus not taking amino acid mixture; Sumanszki et al. [57], mean blood Phe concentration for the 12-month period prior to the study < 600 μmol/L versus > 600 μmol/L

Twelve studies comparing individuals with PKU who adhered to a Phe-restricted diet with a non-adherent population were included in the SLR; however, one study [50] was excluded from the vote-counting analysis because numerical data were not reported (Table 3). There were seven [29, 32, 33, 45, 60–62] studies that reported a higher burden of ≥ 1 comorbidity (or outcome measure) in those who adhered to a Phe-restricted diet compared with those non-adherent (Fig. 5). There were ten studies [5, 32, 33, 45, 47, 54, 60–63] that reported a higher burden of ≥ 1 comorbidity (or outcome measures) in those who did not adhere to a Phe-restricted diet compared with those who adhered to diet.

**Table 3:** Summary of characteristics of studies (*n* = 12) in individuals with PKU adherent to a Phe-restricted diet versus a non-adherent population

Study	Study location	Study design	Age range (years)	Study size, intervention(s), and comparator(s)	Comorbidity types reported	Burden of comorbidity type
Adamczyk et al. 2011 [63]	Europe (Poland)	Cross-sectional study	4.9–21.9	• 45 individuals with PKU on a Phe-restricted diet • 562 reference population (used for Z-score calculation) **Group 1:** individuals with PKU with recommended blood levels of Phe • **Group 2a:** 18 individuals with PKU (Tanner 5 stage) with recommended^a^ blood levels of Phe • **Group 2b:** 12 individuals with PKU (Tanner 5 stage) with blood values of Phe above the recommended level^a^	Bone-related abnormalities	**Significantly lower BMD of the whole skeleton (g/cm**^**2**^ ***p* < 0.01**, **Z-scores *p* < 0.05**,** SD scores *p* < 0.05) and lumbar spine (g/cm**^**2**^ ***p* < 0.0001**, **Z-scores *p* < 0.01, SD scores *p* < 0.05) in PKU group 2b compared with group 2a** No significant difference in BMD Z-scores and SD scores for whole skeleton and lumbar spine between group 1 and 2a^b^
					Musculoskeletal outcomes	**Significantly lower BMC/LBM ratio of the whole skeleton (g/g** ***p* < 0.05**, **SD scores *p* < 0.05) and lumbar spine (g/g** ***p* < 0.01**, **SD scores *p* < 0.05) in PKU group 2b compared with group 2a** Z-score differences between groups were not statistically significant
					Overweight/obesity	Lower bodyweight, bodyweight Z-score, BMI, and BMI Z-score in PKU subgroup 2b compared with subgroup 2a but statistical significance of the difference between groups was not reported
Crujeiras et al. 2015 [45]^c^	Europe (Spain)	Cross-sectional study	0.6–42	• 156 individuals with PKU or mild HPA (unspecified whether all were on a Phe-restricted diet) • 122 individuals with PKU; 18.8% (*n* = 23) treated with BH4 supplementation; 86 with cPKU and 36 with mild or moderate PKU (*n* = 2 and n = 21 treated with BH4 supplementation, respectively) • Reference values used to determine pathologic concentrations of vitamins and minerals	Nutritional outcomes	**Total protein and pre-albumin were significantly lower in those with high adherence to diet compared with those with low adherence (total protein: *p* = 0.0072; pre-albumin: *p* = 0.00011)** **Phosphorus and vitamin B12 were found to be significantly reduced in those with low adherence to diet (phosphorus: *p* = 1.27e**^**−5**^**; vitamin B12: *p* = 0.03)**
Dios-Fuentes et al. 2022 [32]^d^	Europe (Spain)	Retrospective cohort study	Adults 23–38	• 90 individuals with PKU • 43 controlled (Phe < 600 µmol/L) of which 29 on Phe-restricted diet and 14 on sapropterin dihydrochloride • 47 uncontrolled (Phe ≥ 600 µmol/L) of which 43 on Phe-restricted diet and four on sapropterin dihydrochloride	Overweight/ obesity	**Significantly higher median BMI in individuals with uncontrolled Phe levels versus controlled Phe levels in the total population (27.5 [IQR 24.1, 32.8] versus 24.4 [IQR 21.8, 29.4], *p* = 0.023) and in females only (28.1 [IQR 26.0, 33.7] versus 22.6 [IQR 21.2, 28.3], *p* = 0.007);** the difference in median BMI between groups was not statistically significant in males (26.5 [IQR 23.5, 30.3] versus 26.3 [IQR 23.8, 30.0], *p* = 0.923)
					Nutritional outcomes	Numerically lower vitamin B12 concentrations in individuals with controlled Phe levels versus uncontrolled Phe levels (424 pg/mL [IQR 308, 801], versus 530 pg/mL [IQR 419, 751] *p* = 0.347) Higher percentage of individuals with vitamin B12 deficiency in group with controlled Phe levels versus uncontrolled Phe levels, approaching statistical significance (9.3% versus 0%, *p* = 0.053)
Green et al. 2019 [5]	Europe (UK)	Pooled analysis	Adults 16–NR	• 16 individuals with PKU adherent to a Phe-restricted diet • 14 individuals with PKU non-adherent to a Phe-restricted diet • Reference nutrient intake values	Nutritional outcomes	**Significantly higher intake of iron (*p* < 0.001), zinc (*p* < 0.001), vitamin D3 (*p* < 0.001), magnesium (*p* < 0.001), calcium (*p* < 0.001), selenium (*p* < 0.001), iodine (*p* < 0.001), vitamin C (*p* = 0.003), vitamin A (*p* = 0.025), and copper (*p* = 0.004) were reported in individuals who adhered to a Phe-restricted diet compared with those who were non-adherent and reference values** **[*p* values were corrected using an acceptable false discovery rate of 5%]**
						**All amino acids were within normal range of reference values in both groups, except for Phe [*p* values > 0.05]**
Guest et al. 2013 [60]	Europe (UK)	Costing study	> 15–NR	• 94 individuals with PKU (adherent or non-adherent to a Phe-restricted diet) **Group 1:** Remained adherent to a Phe-restricted diet throughout life (*n* = 36) **Group 2:** Individuals with PKU who discontinued a Phe-restricted diet between 15 and 25 years of age (*n* = 22) **Group 3:** Individuals with PKU who discontinued a Phe-restricted diet between 15 and 25 years of age, but restarted the diet at a mean age of 30 years (*n* = 12)	Cardiovascular outcomes	Highest incidence of cardiovascular symptoms in individuals who had never been put on a Phe-restricted diet (group 4) Incidence of cardiovascular symptoms was similar (0.08 and 0.09, respectively) in those who had adhered throughout life (group 1) and in those who had discontinued their diet between 15 and 25 years of age (group 2) None of the group who had discontinued and restarted a Phe-restricted diet experienced cardiovascular symptoms
				**Group 4:** Individuals with PKU who had never been put on a Phe-restricted diet, and still are currently not adherent to any diet (*n* = 14)	Migraine/headache	Highest incidence of headaches in individuals who had discontinued and then restarted a Phe-restricted diet (group 3), followed by those who had never been put on a Phe-restricted diet (group 4), then those who had adhered to a Phe-restricted diet throughout life (group 1) Incidence of headaches was the lowest in those who had discontinued the Phe-restricted diet between 15 and 25 years of age (group 2)
					Cancer	Highest incidence of neoplasia in individuals who discontinued and restarted a Phe-restricted diet (group 3), followed by those who had discontinued their diet between 15 and 25 years of age (group 2) and those who had adhered to a Phe-restricted diet throughout life (group 1) None of the group who had never been put on a Phe-restricted diet had neoplasia
					COPD/asthma	Higher incidence of asthma in individuals who had never adhered to a Phe-restricted diet (group 4) than in those who adhered to a Phe-restricted diet throughout life (group 1) Highest incidence of asthma in individuals who discontinued and restarted a Phe-restricted diet (group 3), followed by those who had never been put on a Phe-restricted diet (group 4) Incidence of asthma in those who had adhered throughout life was the same as in those who had discontinued between the ages of 15 and 25 years
					Gastrointestinal disorders	Highest incidence of gastrointestinal symptoms in individuals who had discontinued and restarted a Phe-restricted diet (group 3), followed by those who had had never been put on a Phe-restricted diet Incidence of gastrointestinal symptoms was the same in those who had adhered to a Phe-restricted diet throughout life as in those who had discontinued their diet between the ages of 15 and 25 years (groups 1 and 2) but lower than that in groups 3 and 4
					Musculoskeletal outcomes	Highest incidence of arthritis/musculoskeletal in individuals who had discontinued and then restarted their Phe-restricted diet (group 3), followed by those who had never been put on a Phe-restricted diet (group 4) The incidence of arthritis/musculoskeletal symptoms was the same in those who had adhered throughout life (group 1) as in those who had discontinued their diet (group 2)
					Dermatologic disorders	Highest incidence of dermatologic symptoms in individuals who discontinued and restarted a Phe-restricted diet (group 3), followed by those who had never been put on a Phe-restricted diet (group 4), then those who had discontinued their diet between 15 and 25 years of age (group 2) Lowest incidence of dermatologic disorders was in those who had adhered to a Phe-restricted diet throughout life (group 1)
					Other	Highest incidence of otolaryngologic and gynecologic symptoms in individuals who had discontinued and then restarted their Phe-restricted diet (group 3), followed by those who had never been put on a Phe-restricted diet (group 4); for those with otolaryngologic symptoms and gynecologic symptoms, those who had discontinued their diet (group 2) The highest incidence of ophthalmologic symptoms was in those who had never been put on a Phe-restricted diet (group 4), followed by those who had discontinued their diet (group 3), and those who had adhered throughout life (group 1) The lowest incidence of otolaryngologic and gynecologic symptoms was in those who had never been put on a Phe-restricted diet (group 4) and the lowest incidence of ophthalmologic symptoms was in those who discontinued and restarted a Phe-restricted diet (group 3)
Koch et al. 2002 [61]	North America (USA)	Cross-sectional study	NR–NR	• Nine individuals with PKU on a Phe-restricted diet past the age of 6 years (adherent group) • 61 individuals with PKU who discontinued a Phe-restricted diet at the age of 6 years (non-adherent group)	Cancer	Both adherent to a Phe-restricted diet and non-adherent groups had zero reports of cancer
					Migraine/headache	Higher percentage of individuals in the non-adherent group reported headaches compared with the adherent group
					Dermatologic disorders	More individuals in the non-adherent group reported eczema compared with the adherent group
					Cardiovascular outcomes	Higher percentage of individuals in the non-adherent group reported heart disease compared with the adherent group
					Hypertension	Higher percentage of individuals in the adherent group reported hypertension compared with the non-adherent group
					COPD/asthma	Higher percentage of individuals in the non-adherent group reported asthma compared with the adherent group
					Overweight/obesity	Higher percentage of individuals in the adherent group reported obesity compared with the non-adherent group
Mazzola et al. 2016 [50]^c^	South America (Brazil)	Cross-sectional study	6–25	• 27 individuals with PKU on a Phe-restricted diet and L-AAM (13 cPKU and 14 mild PKU; 11 early diagnosed and 16 late-diagnosed)	Overweight/obesity	Metabolic control did not affect bodyweight, BMI, body fat mass, or body fat-free mass
Modan-Moses et al. 2007 [29]^d^	Middle East (Israel)	Cross-sectional study	19–41	• 31 individuals with cPKU (adherent and non-adherent to a Phe-restricted diet – number of individuals in each group not specified) • Age- and sex-matched controls used for Z-score calculation	Bone-related abnormalities	Individuals who adhered to a Phe-restricted diet had lower lumbar (*p* = 0.329), femoral neck (*p* = 0.147), and total body (*p* = 0.376) BMD Z-scores compared with non-adherent individuals (results were considered significant if the 2-sided *p* < 0.05; therefore, differences were not statistically significant)
Robinson et al. 2000 [54]^d^	Europe (UK)	Cross-sectional study	11–38	• 83 individuals with PKU (22 on a strict diet, 30 on a relaxed diet, 31 on an unrestricted diet) • Reference population for mean blood vitamin B12 concentration and erythrocyte folate concentration (normal population data for Vitamin B12 obtained from the Office of Population Censuses and Surveys [The Dietary and Nutritional Survey of British Adults])	Nutritional outcomes	Individuals with PKU who adhered to a restricted diet had comparable vitamin B12 concentrations to the reference population whereas individuals on a relaxed or unrestricted diet had significantly lower vitamin B12 concentrations compared to the reference population (*p* = 0.0034 and *p* < 0.0001, respectively)
Rojas-Agurto et al. 2023 [33]^d^	South America (Chile)	Retrospective cohort study	Adults 18.5–27	• 24 individuals with PKU who began a Phe-restricted diet from neonatal diagnosis	Musculoskeletal outcomes	There were no significant differences in left- or right-handgrip strength between the PKU-1 and PKU-2 groups
				• PKU-1 group: 10 individuals who continued on Phe-restricted diet with adequate supply of protein substitute and kept strict follow-up	Bone-related abnormalities	There were no significant differences in spine or femoral BMD between the PKU-1 and PKU-2 groups
				• PKU-2 group: 14 individuals who discontinued protein substitute and micronutrient supplementation at 18 years of age and stopped attending metabolic control appointments^e^	Overweight/obesity	Although individuals in the PKU-2 group tended to have higher bodyweight, WC, BMI, total fat mass, and lower total fat-free mass and appendicular fat-free mass than those in the PKU-1 group, differences between groups were not statistically significant
					Nutritional outcomes	**Significantly lower mean vitamin B12 concentration in the PKU-2 group compared with the PKU-1 group (338 ng/mL [IQR 137, 539] versus 722.5 ng/mL [IQR 499, 926], *p* = 0.03)** **Significantly lower mean vitamin D3 concentration in the PKU-2 group (below reference) compared with the PKU-1 group (23.9 pg/mL [IQR 24.8, 32.3] versus 35.7 pg/mL [IQR 28.4, 46.6], *p* < 0.01)** **Significantly lower mean intake of vitamin B12 (*p* < 0.01), vitamin D (*p* = 0.02), and calcium (*p* = 0.01) in the PKU-2 group compared with the PKU-1 group** Differences between groups in serum folic acid and homocysteine were not statistically significant and levels were within range in both groups The difference between groups in intake of folic acid was not statistically significant
Schulz et al. 1995 [62]	Europe (Germany)	Cross-sectional study	12–29	• 99 individuals with PKU (82 adherent and 17 non-adherent to a Phe-restricted diet)	Nutritional outcomes	**Male individuals with PKU who adhered to a Phe-restricted diet had a significantly lower intake of vitamin B12 compared with non-adherent individuals with PKU (*p* < 0.05)** **Female individuals with PKU who adhered to a Phe-restricted diet had a significantly higher intake of vitamin B12 compared with non-adherent individuals with PKU (*p* < 0.05)**
Sumanszki et al. 2019 [57]^d^	Europe (Hungary)	Cross-sectional study	18–49	• 69 individuals with PKU on a Phe-restricted diet and/or AAM • Individuals were grouped based on their adherence to a low-protein diet (30 in low-adherence group and 37 in good-adherence group) and AAM consumption (25 in reduced AAM-intake group and 42 in adequate AAM-intake group) • 50 healthy controls	Other^f^	**Serum TSH was significantly different between the 3 groups (low adherence, good adherence, and control, *p* = 0.006). Post-hoc test showed significantly lower serum TSH in the low adherence group compared with the good adherence (*p* = 0.018) and control (*p* < 0.001) groups** **Median UIC (*p* < 0.001) and UIC/Cr (*p* < 0.001) were significantly different between the three groups (low adherence, good adherence, and control, *p* = 0.006). Post-hoc tests indicated significantly lower UIC in the low-adherence group compared with the good-adherence (*p* < 0.001) and control (*p* < 0.001) groups. UIC/Cr was significantly higher in the good-adherence group compared with the low-adherence (*p* < 0.001) and control (*p* = 0.001) groups** No significant differences were found in thyroid volume (*p* = 0.603) and in thyroid nodule incidence (*p* = 0.157) after stratifying based on adherence to a low-protein diet

Results in bold font showed a statistically significant difference between groups

*AAM* amino acid mixture, *BH4* tetrahydrobiopterin, *BMC/LBM* bone mineral content/lean bone mass, *BMD* bone mineral density, *BMI* body mass index, *COPD* chronic obstructive pulmonary disease, *cPKU* classical PKU, *IQR* interquartile range, *L-AAM* L-amino acid mixture, *NR* not reported, *Phe* phenylalanine, *PKU* phenylketonuria, *SD* standard deviation, *TSH* thyroid-stimulating hormone, *UIC* urinary iodine concentration, *UIC/Cr* creatinine-normalized urinary iodine concentration

^a^The recommended blood Phe levels in treated individuals < 12 years old is 2–6 mg/dL and 2–10 mg/dL for individuals ≥ 12 years old, whereas the level for healthy people is < 2.8 mg/dL

^b^Authors conducted statistical testing and reported that the difference between groups was not significant but did not report the *p* value

^c^Study appears in Tables 2, 3, and 5

^d^Study appears in both Tables 2 and 3

^e^Three individuals in the PKU-2 group admitted occasional transgressions in the diet and ate meat products (chicken, tuna, egg), included in one of two record days. The remaining eleven maintained a vegan diet without transgressions

^f^ Thyroid function

Six studies investigating nutritional outcomes were included in the analysis of adherent versus non-adherent PKU populations [5, 32, 33, 45, 54, 62], with five studies reporting a higher burden of ≥ 1 outcome measure in those who were non-adherent [5, 33, 45, 54, 62] and three reporting a higher burden in those who were adherent [32, 45, 62]. In one study [45], the impact of dietary adherence differed with respect to the nutritional outcome measured. Total protein and serum pre-albumin concentrations were significantly lower in those with high adherence to diet versus those with low adherence (*p* = 0.0072 and *p* = 0.00011, respectively), whereas concentrations of phosphorus and vitamin B12 were significantly lower in those with low adherence versus those with high adherence (*p* < 0.0001 and *p* = 0.03, respectively) [45]. A second study [62] reported lower intake of all vitamins and minerals measured (statistically significant differences, except for potassium and phosphorus) in those not taking amino acid mixture (AAM) compared with those who adhered to AAM, except for intakes of vitamin B12 and niacin in males, which were higher in those not taking AAM, but the difference was only statistically significant for vitamin B12). Significantly lower intakes of many micronutrients were reported in non-adherent compared with adherent groups in a third study [5]; however, intakes of manganese, potassium, vitamin B12, sodium, chloride, and folate were similar between groups. In a fourth study [33], significantly lower (below reference levels) serum vitamin D3 (*p* < 0.01) and vitamin B12 (*p* = 0.03) were reported in individuals who had discontinued the protein substitute at 18 years of age and stopped attending metabolic control appointments (non-adherent group) compared with those who had continued adherence to a Phe-restricted diet with an adequate protein substitute (above reference levels). Levels of folic acid were lower and homocysteine levels were higher in the non-adherent group compared with the adherent group (but the difference between groups was not statistically significant and levels of both nutrients in both groups were within the reference range).

Although concentrations of vitamin B12 and folate measured in a fifth study [54] were lower in the unrestricted diet group than in the strict low-Phe diet group, statistical significance was only assessed versus the control population. In the sixth study [32], vitamin B12 levels were higher in the group of individuals with uncontrolled Phe levels (non-adherent) than in the group with controlled Phe levels (adherent) and this group included a higher percentage of individuals with vitamin B12 deficiency that was almost statistically significant (*p* = 0.053).

Four studies [33, 60, 61, 63] investigated several types of comorbidities in diet-adherent versus non-adherent populations, including cardiovascular outcomes, migraine/headaches, cancer, COPD/asthma, and dermatologic outcomes [60, 61], overweight/obesity [33, 61, 63, 64], musculoskeletal outcomes [33, 60, 63], bone-related abnormalities [33, 63], hypertension [61], gastrointestinal outcomes, and other outcomes [60]. The direction of the higher burden for several comorbidity types differed between studies or between different subgroups within the same study.

A higher burden of cardiovascular [60, 61], dermatologic [60, 61], migraine/headaches [60, 61] and other [57, 60] outcomes was found in non-adherent compared with adherent populations, in two studies each, compared with a higher burden for these same comorbidity types in adherent compared with non-adherent populations, in one study of migraine/headaches [60] and other outcomes [60], and no studies of cardiovascular or dermatologic outcomes. A higher burden of COPD/asthma was found in the non-adherent versus adherent population in two studies (see Fig. 5 footnotes [60, 61]), while a higher burden of COPD/asthma was found in an adherent versus non-adherent population in one of these studies (see Fig. 5 footnotes [60]).

A higher burden of hypertension [61] was found in those who adhered to a Phe-restricted diet compared with those who were non-adherent. No studies reported a higher burden of hypertension in those who did not adhere. Another study [32] reported the prevalence of hypertension among the overall population of individuals with PKU as 7.9%, but the prevalence in the subgroups with controlled and uncontrolled Phe levels was not reported.

There was one study [63] with a higher burden of bone-related abnormalities in those with uncontrolled Phe levels (classified as non-adherent in the vote-counting analysis) compared with those with controlled Phe levels (considered as adherent in the vote-counting analysis) and two studies [29, 33] with a higher burden in those who were adherent compared with those who were non-adherent.

The direction of the higher burden for overweight/obesity differed between studies. In one study [32], BMI was significantly higher in the total population and in women with uncontrolled Phe levels than in those with controlled Phe levels; median BMI for the total population was 27.45 kg/m^2^ versus 24.36, *p* = 0.023; median BMI for the female population was 28.11 versus 22.58, *p* = 0.007, but the difference in median BMI between men with controlled Phe levels and those with uncontrolled Phe levels was not statistically significant (*p* = 0.923). It should be noted that 18/90 (20%) of included individuals in the study received BH4 rather than dietary therapy, and eight of these required Phe-free amino acid formula to achieve metabolic control [32]. Two studies [61, 63], reported a higher burden of overweight/obesity in individuals who were diet-adherent [61] or had controlled Phe levels [63] compared with the non-adherent [61] or uncontrolled Phe levels [63] group: 33.3% versus 16.4% with obesity [61] and higher bodyweight and BMI (absolute and Z-scores) [63] but statistical significance of the difference between groups for individual comorbidities was not assessed. In a third study [33], there were numerical increases in bodyweight, WC, BMI, and total fat mass, and numerical increases in appendicular fat-free mass in the diet-adherent group compared with the group who had discontinued the Phe-restricted diet at 18 years of age (measures of total fat-free mass were similar) but differences between groups were not statistically significant. An additional study [50] found no effect of metabolic control on BMI classification and bioelectrical impedance parameters (indicators of overweight/obesity) but numerical data were not reported, hence this study could not be included in the vote-counting analysis.

The burden of gastrointestinal symptoms and cancer was higher in adherent compared with non-adherent populations in one study [52], but also higher in non-adherent compared with adherent populations for these same comorbidities. This study [52] compared groups with varying levels of adherence: the highest incidence of gastrointestinal symptoms was in those who had never been treated with a Phe-restricted diet, was similar in those who had adhered to dietary treatment throughout life and in those who had discontinued dietary treatment between the ages of 15 and 25 years [60]. None of the group who had discontinued and restarted a Phe-restricted diet experienced gastrointestinal symptoms [60]. Conversely, the incidence of cancer was highest in those who had discontinued and restarted a Phe-restricted diet, followed by those who had discontinued their diet between 15 and 25 years of age and then those who had adhered to a Phe-restricted diet throughout life. None of the group who had never been treated with dietary therapy had cancer [60].

#### Individuals with PKU on different Phe-restricted diets

A comparison of somatic comorbidities in individuals with PKU on different Phe-restricted diets was reported in four studies (Table 4). These studies did not compare a PKU population on a Phe-restricted diet with either healthy controls or no intervention, and therefore did not meet the inclusion criteria of the SLR. The scenario of those on different diets being compared was not anticipated by the PICOS criteria but nevertheless these studies have been included because the comparison is of potential interest, from the perspective of the impact of dietary improvements on the clinical burden of somatic comorbidities.

**Table 4:** Summary of characteristics of studies (*n* = 4) comparing individuals with PKU on different Phe-restricted diets

Study	Study location	Study design	Age range (years)	Study size, intervention(s), and comparator(s)	Comorbidity types reported	Burden of comorbidity type
Das et al. 2014 [67]	Europe (Germany)	Cross-sectional study	Adults 16–44	• 51 individuals with PKU^a^ Individuals were either not on diet (normal food group), on a vegan diet without AAM (vegan group), on a vegan diet with AAM (vegan group + AAM) or on a protein reduced diet with AAM supplements (PKU diet)	Nutritional outcomes	No statistical differences were found in trace elements such as iron, zinc, or selenium between groups (no *p* value provided). However, there was one individual who returned to the clinic in response to the invitation for the study after many years without monitoring who had reduced levels of selenium and zinc
MacDonald et al. 2004 [66]	Europe (UK)	Open intervention trial	8–37	• 23 individuals with PKU (Pre-trial Phe-restricted diet vs new Phe-restricted diet) • Dietary reference values (nutrients) The pre-trial Phe-restricted diet had a protein substitute which included significant quantities of added carbohydrate. The new Phe-restricted diet was a low-carbohydrate Phe-free protein substitutes	Nutritional outcomes	**Significant increase in selenium (*p* < 0.001), calcium (*p* < 0.01), and zinc (*p* < 0.001) intake in individuals with PKU on the new Phe-restricted diet compared with the pre-trial Phe-restricted diet**
						On the new Phe-restricted diet, all nutrients exceeded dietary reference values
					Overweight/obesity^b^	BMI remained unchanged between individuals on the pre-trial Phe-restricted diet versus the new Phe-restricted diet
Pena et al. 2021 [64]	Europe (Portugal)	Retrospective cohort study	15–43	• 11 individuals with PKU on a Phe-restricted diet (Blood pressure and weight measurements *n* = 11, body fat measurement *n* = 9) (Individuals received L-AA for up to 12 months and then switched to CGMP-AA for a mean length of time of 29 months [range 6–48 months])	Overweight/obesity	Individuals on CGMP-AA had a tendency for increased body weight (*p* = 0.064) and total body fat (*p* = 0.056) when compared with baseline L-AA
						However, the overall percentage of overweight and obesity in individuals on L-AA versus CGMP-AA remained unchanged (*p* = 1.000)
					Cardiovascular outcomes	There were no statistical differences in blood pressure between individuals on L-AA versus CGMP-AA (systolic blood pressure [*p* = 1.000], diastolic blood pressure [*p* = 0.581], and heart rate [*p* = 0.607])
Pinto et al. 2017 [65]	Europe (Portugal)	Retrospective case series	13–42	• 11 individuals with PKU or HPA (pooled) on a Phe-restricted diet [6 cPKU, 4 mild PKU, 1 HPA] (Individuals received L-AA for up to a median of 12 months and then switched to GMP-AA for a median > 12 months)	Diabetes	**Hemoglobin A**_**1C**_** decreased in individuals after introduction of the GMP substitute (*p* = 0.011)**
					Overweight/obesity	Anthropometry and body composition analysis measurements were similar between individuals on the GMP substitute and those on L-AA supplements, with no changes in the prevalence of overweight and obesity (BMI *p* = 0.367)
					Cardiovascular outcomes	Blood pressure remained the same after the introduction of GMP

Results in bold font showed a statistically significant difference between groups

*AAM* amino acid mixture, *BMI* body mass index, cPKU classical PKU, *CGMP-AA* modified casein glycomacropeptide supplements, *GMP* glycomacropeptide-based protein substitute, *GMP-AA* glycomacropeptide-based protein amino acid, *HPA* hyperphenylalaninemia, *L-AA* L-amino acid, *Phe* phenylalanine, *PKU* phenylketonuria

^a^Number of individuals in each treatment group is not reported

^b^Authors did not report outcome values for controls therefore, results are not comparative

Two studies [64, 65] compared L-amino acid (L-AA) supplements versus glycomacropeptide-based (GMP) protein substitute (GMP-AA) or modified casein GMP amino acid (CGMP-AA) supplements; one study [66] compared a pre-trial Phe-restricted diet (protein substitute that included significant quantities of added carbohydrate) with a new Phe-restricted diet (low-carbohydrate Phe-free protein substitutes); another study [67] compared individuals not on a diet (normal food group) versus those on a vegan diet without AAM versus those on a vegan diet with AAM versus a protein reduced diet with AAM supplements.

One study [65] reported no changes in the prevalence of overweight/obesity (BMI *p* = 0.367); another study [64] reported a tendency for increased body weight (*p* = 0.064) and total body fat (*p* = 0.056) in individuals on CGMP-AA when compared with baseline on L-AA, but these changes were not statistically significant. Two studies [64, 65] reported on cardiovascular outcomes, and no differences in blood pressure between those on L-AA versus those on GMP-AA or CGMP-AA were found. One study [67] reported on nutritional outcomes and found no statistically significant difference was found in trace elements iron, zinc, or selenium between those not on a diet versus a vegan diet without AAM versus a vegan diet with AAM versus a protein reduced diet with AAM (no *p* value reported).

#### Individuals on a Phe-restricted diet with more severe PKU versus those with HPA or less severe PKU

Five studies reported on somatic comorbidities experienced by individuals on a Phe-restricted diet with cPKU (more severe form of disease) versus individuals with HPA or less severe PKU (Table 5). One study [68] reported bone-related abnormalities in individuals with cPKU versus those with mild or moderate PKU; one study [55] reported anthropometric parameters and markers of metabolic syndrome/diabetes in individuals with cPKU, mild or moderate PKU, and mild HPA; one study [50] reported BMI classifications (relating to underweight, normal weight, overweight, and obese) and bioelectrical impedance parameters (relating to fat mass and fat-free mass) in individuals with cPKU and mild PKU; one study [69] reported nutritional outcomes in individuals with PKU versus those with HPA; and one study [45] reported nutritional outcomes in individuals with cPKU, mild or moderate PKU, and HPA (Table 5).

**Table 5:** Summary of characteristics of studies (*n* = 5) in individuals with more severe PKU on a Phe-restricted diet versus individuals with HPA or less severe PKU on a Phe-restricted diet

Study	Study location	Study design	Age range (years)	Study size, intervention(s), and comparator(s)	Comorbidity types reported	Burden of comorbidity type
Couce et al. 2018 [44]	Europe (Spain)	Cross-sectional study	NR	• 33 individuals with PKU on a relaxed Phe-restricted diet ◦ 57 (68.7%) had PKU and 26 (31.3%) had mild HPA ◦ Of those with PKU, 37 (64.9%) had cPKU and 20 (35.1%) had mild or moderate PKU ◦ One individual with cPKU and nine individuals with mild or moderate PKU also received BH4 • 68 healthy controls	Overweight/obesity	**Compared with the mild HPA group, a significantly higher proportion of individuals in the PKU group had BMI and WC above the upper limit (87.5% versus 12.5%;** ***p*** **= 0.0062)** **When focusing only on adults, there was also a significantly higher proportion of individuals in the PKU group with BMI and WC above the upper limit compared with the HPA group (68.8% versus 21.3% for BMI; *p* = 0.010, and 71.9% versus 28.1% for WC; *p* = 0.0011)**
					Diabetes	**HOMA-IR was significantly lower in individuals with HPA compared with those with PKU (*p* = 0.034)** **Comparison between individuals with cPKU, mild or moderate PKU, and HPA revealed a positive correlation between disease severity and HOMA-IR value (*p* = 0.037)**
						**Quick index value was significantly higher in individuals with HPA compared with those with PKU (*p* = 0.019)** **Fasting insulin levels above the upper limit were reported in a significantly higher proportion of those with PKU versus HPA (*p* = 0.035)** **Comparison between individuals with cPKU, mild or moderate PKU, and HPA revealed a negative correlation between disease severity and Quick index score (*p* = 0.026)**
Crujeiras et al. 2015 [45]^a^	Europe (Spain)	Cross-sectional	10.2 months–42	• 122 individuals with PKU; 18.8% (*n* = 23) treated with BH4 supplementation; 86 with cPKU and 36 with mild or moderate PKU (*n* = 2 and n = 21 treated with BH4 supplementation, respectively) • Reference values used to determine pathologic concentrations of vitamins and minerals	Nutritional outcomes	**Individuals with mild HPA or mild or moderate PKU had significantly lower vitamin B12 and zinc blood concentrations compared with individuals with cPKU (vitamin B12: *p* = 0.0046; zinc: *p* = 0.03)** **Pre-albumin, ferritin, and folic acid concentrations were found to be diminished in individuals with mild HPA compared with those with PKU (ferritin: *p* = 0.0084; Pre-albumin: *p* = 0.024; folic acid: *p* = 0.0147)** **Selenium and phosphorus were found to be reduced in PKU versus Mild HPA (selenium: *p* = 0.0034; phosphorus: *p* = 0.0056)**
Mazzola et al. 2016 [50]^a^	South America (Brazil)	Cross-sectional study	6–25	• 27 individuals with PKU on a Phe-restricted diet and L-AAM (11 early diagnosed and 16 late diagnosed) • 13 cPKU • 14 mild PKU	Overweight and obesity	No statistically significant difference in bodyweight, BMI, body fat mass, and body fat-free mass between mild PKU and cPKU
Mirás et al. 2013 [68]	Europe (Spain)	Cross-sectional study	Adults 27.1–41	• 43 individuals with PKU on a Phe-restricted diet • (27 with cPKU, nine with moderate PKU, seven with mild PKU)	Bone-related abnormalities	Prevalence of osteopenia and osteoporosis was similar between adults with cPKU and those with mild or moderate PKU. Out of the four adults with PKU that had MBD, *n* = 2 had osteoporosis (one cPKU and one moderate PKU), and *n* = 2 had osteopenia (one cPKU and one moderate PKU)
Procházková et al. 2013 [69]	Europe (Czech Republic)	Cross-sectional study	1–42	• 51 adults with PKU on a low-protein diet and receiving Phe-free amino acid mixtures • 10 adults with HPA on a low-protein diet and/or receiving Phe-free amino acid mixtures	Nutritional outcomes	The differences in the concentrations of serum pre-albumin (*p* = 0.573), zinc (*p* = 0.652), and iron (*p* = 0.853) among the respective groups were not statistically significant. The median of serum pre-albumin and iron in all the examined groups reached the normal reference values in our laboratory A statistically significant difference in concentrations of serum selenium was found between PKU and HPA individuals in adulthood (*p* = 0.006). The median of serum selenium values was reduced in all PKU and HPA children. The median of serum selenium was also reduced in PKU adults

*BH4* tetrahydrobiopterin, *BMI* body mass index, *cPKU* classical PKU, *HOMA-IR* Homeostatic Model Assessment for Insulin Resistance, *HPA* hyperphenylalaninemia, *L-AAM* L-amino acid mixture, *MBD* mineral bone disease, *NR* not reported, *Phe* phenylalanine, *PKU* phenylketonuria, *WC* waist circumference

^a^Study appears in Tables 2, 3, and 5

In the study comparing cPKU with mild or moderate PKU [68], the prevalence of osteopenia and osteoporosis was reported to be similar between those in either group on a Phe-restricted diet; statistical significance for the difference between groups was not reported.

One study [44] reported BMI and WC above the upper limit (indicating overweight/obesity) in a significantly higher proportion of individuals with PKU versus mild HPA (*p* = 0.0062 for overall population; *p* = 0.010 for BMI and *p* = 0.0011 for WC in adults) while another study [50] found that type of PKU (cPKU or mild PKU) did not affect BMI classifications or bioelectrical impedance parameters (numerical data were not reported). Fasting insulin levels above the upper limit were reported in a significantly higher proportion of those with PKU versus mild HPA (*p* = 0.035). Homeostatic Model Assessment for Insulin Resistance (HOMA-IR) was significantly increased (*p* = 0.034) and Quick index score was significantly decreased (*p* = 0.019) in those with PKU versus mild HPA, both indicating worse insulin resistance in those with more severe forms of the disease. Quick index score was significantly lower, and HOMA-IR was significantly higher, in patients with cPKU than in those with mild or moderate PKU and those with mild HPA; therefore increasing severity was correlated with increasing BMI, WC, and age [44].

In one study [69], no statistically significant differences were found in the concentrations of serum pre-albumin, zinc, and iron between adults with PKU and those with HPA (*p* value not provided) [69]. However, there was a statistically significant lower concentration of selenium in adults with PKU compared with adults with HPA (*p* = 0.006). Another study [45] found that concentrations of selenium and phosphorous were significantly reduced in those with PKU (mild/moderate and cPKU grouped together) versus mild HPA (*p* = 0.0034 and *p* = 0.0056, respectively), although there were only five individuals with phosphorous levels lower than the normal limit. Conversely, serum pre-albumin, ferritin, and folic acid concentrations were significantly reduced in those with mild HPA versus those with mild, moderate, or cPKU (*p* = 0.024, *p* = 0.0084, and *p* = 0.0147, respectively) [45]. In the same study, vitamin B12 and zinc were significantly reduced in those with mild HPA and mild or moderate PKU compared with those with cPKU (*p* = 0.0046 and *p* = 0.03, respectively) [45]. However, it should be noted that levels of total protein, calcium, phosphorous, vitamin B12, ferritin, and zinc were within the normal range in the majority of individuals with PKU, and none had a folic acid deficiency [45].

## Discussion

### Main findings

This review has highlighted the breadth of somatic comorbidities experienced by individuals with PKU, and the higher clinical burden versus a non-PKU population. The findings add to the published literature, confirming the comorbidity burden in individuals with PKU treated with a Phe-restricted diet [26, 70, 71]. The most commonly reported somatic comorbidities in studies of individuals with PKU on a Phe-restricted diet with or without pharmacologic therapy compared with healthy controls or reference values were bone-related abnormalities [15, 16, 22, 24–42], followed by overweight/obesity [15, 16, 22, 33, 43, 44, 49–54], nutritional outcomes [22, 26, 33, 45, 48, 49, 54, 56, 58, 59], and cardiovascular outcomes [15, 22, 43, 46, 47]. It was not possible to draw definitive conclusions from the other three population comparisons due to the limited number of studies included in each comparison and differences in the comorbidity types covered: adherent to a Phe-restricted diet versus non-adherent, twelve studies [5, 29, 32, 33, 45, 50, 54, 60–63]; groups on different Phe-restricted diets, four studies [64–67]; and more severe PKU versus HPA or less severe PKU, five studies [44, 45, 50, 68, 69].

### Relation of main findings to prior research

In a published SLR, Pessoa et al*.* reported a high prevalence of clinical complications (e.g., overweight/obesity and osteopenia), poor adherence to clinical recommendations, negative socioeconomic impact, and negative impact on caregivers of Latin American patients with PKU of all ages (diagnosed with PKU within the first 3 months of life) [70]. The study concluded that dietary management alone was not sufficient to prevent the burden of PKU, which concurs with the findings of our SLR, in which individuals with PKU were compared with healthy controls or a reference population in the vote counting analysis. It is important to acknowledge that our SLR did not investigate the negative socioeconomic impact, or the negative impact on caregivers.

#### Individuals with PKU on a Phe-restricted diet with or without pharmacologic therapy versus healthy controls or reference values

Abnormal bone status has been a long-standing concern in individuals with PKU [3, 72]; therefore, it is not surprising that the most reported somatic comorbidities in individuals with PKU on a Phe-restricted diet in our SLR were bone-related abnormalities. BMD Z-score was the most reported outcome measure, but a variety of other outcome measures was reported among the included studies and there was a higher burden of at least one bone-related outcome in individuals with PKU compared with healthy (non-PKU) controls in many of these studies. Currently, it is unclear whether low BMD in individuals with PKU is a direct consequence of the disease, a complication of following a Phe-restricted diet or due to reliance on low-Phe amino acid supplementation (medical foods), which can increase urinary calcium and magnesium excretion [28, 38, 73]. Emerging evidence suggests that the PKU population may be at increased risk of metabolic acidosis, which has been linked to low bone mineralization [74]. This adds to the debate on whether the increased renal acid load from consumption of low-Phe medical foods is related to low BMD and highlights the need to further explore the etiology and impact of bone-related abnormalities in individuals with PKU. A meta-analysis has been conducted to investigate BMD outcome measures in adults with PKU, and to explore the impact of the Phe-restricted diet (including the impact of adherence to diet) on BMD. For further details on the BMD meta-analysis and its findings, refer to the separate meta-analysis publication.

Burton et al. [16] reported that high blood Phe levels may impact biological mechanisms that are related to increased risk of comorbid conditions such as obesity, renal disease, metabolic dysfunction, and cardiovascular complications, which might explain why nutritional outcomes, cardiovascular outcomes, and overweight/obesity were also commonly reported in the studies included in our SLR.

The Phe-restricted diet limits the intake of natural protein to vegetable sources, and despite the availability of low-Phe medical foods, a significant number of adolescents and adults do not consume adequate amounts of protein substitutes [58]. As a result, individuals with PKU have been shown to be at risk of deficiencies in nutrients such as carnitine and vitamin B12, which are derived from animal protein sources [49, 54, 58, 59]. Clinical symptoms of carnitine deficiency include muscle weakness or cardiomyopathy, which may be caused by low intake of dietary carnitine, deficient synthesis of carnitine, or acyl-carnitine production [59]. Vitamin B12 deficiency may lead to anemia, gastrointestinal, and neurological symptoms [58].

Folate is an essential vitamin that plays a crucial role in metabolism [75]. High levels of folate have been attributed to the high folic acid content in protein substitutes [76]; however, higher and lower concentrations of folate in individuals with PKU compared with controls (as well as levels above or within the normal range) have been reported [26, 33, 45, 48, 54, 76]. For both vitamin B12 and folate, the risk of deficiency was higher in those who were not following a strict low-Phe diet with adequate amino acid and vitamin and mineral supplementation [45, 54]. Hochuli et al. [77] also found that a relaxation of AAM intake resulted in insufficient nutrient supply despite a compensatory increase in consumption of natural protein. The evidence from these studies indicates the need for continual dietary guidance through adulthood, as inadequate intake of nutrients can lead to further comorbidities. One study included in our SLR [46] reported an association between high Phe levels and arterial stiffness, which impacts the risk of cardiovascular disease [16]. However, another included study [47] did not identify any significant difference in arterial stiffness or carotid intima media thickness (a surrogate marker of atherosclerosis) compared with healthy controls. Similar to other comorbidities, there are limited data available to explain whether an increased cardiovascular risk in individuals with PKU is due to the disease itself or factors related to the Phe-restricted diet [32].

Given the potential for increased risk of obesity with high blood Phe levels noted by Burton et al. [16], we felt it was important to acknowledge the inconsistency among conclusions of the studies reporting this outcome that were included in our SLR and other previously published SLRs. Of the studies included in our SLR, four studies [33, 50, 53, 55] found no significant difference in the proportions of individuals with PKU who were overweight or obese (as measured by prevalence, body weight, WC, BMI, body fat percentage, total or appendicular body fat mass, total or appendicular fat-free mass, or central obesity) compared with healthy controls; however, a significantly higher BMI, prevalence ratio or percentage of individuals with overweight/obesity in individuals with PKU compared with matched controls was reported in four studies [15, 16, 22, 43]. Two studies provided supporting evidence for an increased burden of overweight/obesity in individuals with PKU: in one study [44], there was a higher proportion of overweight/obesity in those with PKU versus controls, but statistical significance of the difference between groups was not reported; and in another study [52] there was a higher rate of obesity among females with PKU in four of six centers, but the overall proportion of overweight individuals was lower in five of the six centers studied. The results of the tenth study [56] were conflicting, with higher percentages of overweight and lower percentages of obese individuals in the PKU group versus controls (statistical significance was not reported). Two published SLRs identified in our review have also reported conflicting results on the prevalence or risk of overweight/obesity in individuals with PKU [78]: one SLR, published in 2021 [71], concluded that individuals with PKU (including children, adolescents, and adults) had similar BMI to healthy controls, although BMI was significantly higher than healthy controls in a subgroup of individuals with cPKU; another SLR, published in 2023 [78], concluded that adults with PKU had a higher BMI and higher prevalence of obesity compared with a matched control population but the proportions of the PKU population with obesity varied between studies from 4.5% to 72% and the findings were inconsistent when compared with the general population. A previously reported SLR and meta-analysis investigating whether a Phe-restricted diet is a risk factor for overweight/obesity in individuals with PKU found that BMI was similar between individuals with PKU and healthy controls [71]. In the study reporting the frequency of overweight/obesity in individuals with PKU receiving different treatments [56], the highest percentage of overweight individuals was in the dietary therapy group, followed by the pegvaliase group and sapropterin dihydrochloride group, but the highest percentage of obese individuals was in the pegvaliase group and the lowest was in the dietary therapy group; therefore, the pegvaliase group had the highest rate of overweight/obesity overall and the sapropterin dihydrochloride group had the lowest.

Differences in population characteristics relevant to obesity may have contributed to the different outcomes observed between studies included in our SLR, e.g., PKU cohorts in studies with no significant difference from controls tended to include younger participants (mean age 14.4 [55], 23.5 [33], and 26.0 [53] years, mean age not reported [range 6–25 years] [50] versus mean age 30.8 [43], 34.6 [16], 41.2 [22], and 50.9 [15] years) and in three studies [16, 22, 43], were all early-diagnosed/treated, as opposed to Trefz et al. [15], who included a higher number of late- versus early-diagnosed participants (*n* = 216 versus *n* = 161) [15, 53, 55]. A study comparing early- versus late-diagnosed individuals found that the proportion of those with a BMI above the upper limit was almost twice as high in late- versus early-diagnosed participants (*p* = 0.023) [44]. However, timing of diagnosis was not reported in two studies [16, 43] and the proportion of late-diagnosed individuals was relatively low (29.7%) in another study [22]. Differences in male:female ratio may also play a role, as studies reporting a significant difference in the prevalence or risk of overweight/obesity tended to include a higher proportion of females with PKU than studies finding no significant difference between groups (46% [55], 48.1% [50], 50% [33], and 51% [53] female versus 56% [22], 58.1% [15], and 63.7% [16] female). These results are supported by a published SLR, which found that overweight/obesity was 2–3 times more frequent in females with PKU than males [78], and the results of Ozel et al. [52] included in our SLR. Azabdaftari et al. [43] was the exception, reporting significantly higher BMI in adults with PKU compared with healthy controls, of whom only 39% were female. Furthermore, Rocha et al. [55] found no effect of male:female ratio on prevalence of overweight/obesity. Overweight/obesity is a complex comorbidity that is likely to be impacted by the components of the Phe-restricted diet, adherence to diet, and other factors relating to individual patient behaviors. The inconsistency in findings indicates a need for further research.

#### Individuals with PKU adherent to a Phe-restricted diet versus a non-adherent population

Adherence to a Phe-restricted diet is often determined by using blood Phe levels as an indicator (where low blood Phe levels indicate a greater level of treatment adherence). However, it is important to note that blood Phe levels are also dependent on the extent of an individual’s functional PAH, which in turn is determined by the mutations in the gene encoding PAH for that individual [79, 80]. An individual’s genotype correlates with biochemical phenotype, including the degree of Phe tolerance linked to disease severity [80], and so elevated blood Phe levels in some treated individuals (e.g., those with mutations rendering PAH inactive), may be due to low Phe tolerance and severe disease rather than non-adherence. A recent study, included in the SLR, comparing maintenance and suspension of dietary treatment in adults with PKU noted that an elevated blood Phe level, as a result of abandoning a Phe-restricted diet, has the potential to affect muscle and bone health, but other factors such as reduced quality of protein intake, reduced intake of vitamins and minerals, and lower physical activity, could also play a part [33]. Further investigation on the impact of adherence to diet on the clinical burden of somatic comorbidities is needed due to the limited number of studies currently published.

#### Individuals with PKU on different Phe-restricted diets

As part of MNT, individuals with PKU can be placed on different types of Phe-restricted diets and/or protein-based supplements. Elevated blood Phe levels can lead to serious nutritional deficiencies, and studies have reported that individuals who did not adhere to a Phe-restricted diet had higher blood Phe levels in comparison with those following a Phe-restricted diet [67]. Despite this, the studies in our SLR reported that many adults do not adhere to dietary recommendations owing to the taste, smell, and texture of protein substitutes/supplements and the inconvenience of these diets [66, 67].

It has also been suggested that the Phe-restricted diet may be linked to certain comorbidities. For example, a recent SLR [78] found that low-Phe food substitutes tend to be higher in carbohydrates, which is a known causative factor for overweight and obesity. However, a previous study found that BMI only increased in 55% of individuals receiving lower versus higher carbohydrate Phe-free protein substitutes; was unchanged in 5%; and actually decreased in 40%; and, overall, the difference between groups was not statistically significant [66]. Additionally, studies that compared the prevalence of obesity between individuals receiving amino acids versus GMP substitute reported conflicting results [64, 65].

#### Individuals on a Phe-restricted diet with more severe PKU versus those with HPA or less severe PKU

Poor metabolic control has previously been reported in individuals with cPKU receiving dietary treatment compared with individuals with mild PKU or HPA [6]. Despite the extensive amount of research into the importance of metabolic control, there is limited understanding of the impact of PKU severity on the clinical burden of somatic comorbidities.

In our SLR, a study that assessed individuals with mild, moderate, and cPKU found no relationship between PAH genotype and the development of mineral bone disease (MBD), no difference in the blood Phe levels between individuals with PKU who developed MBD and those who did not, and no relationship between diet compliance and MBD [68]; however, individuals with osteopenia or osteoporosis in this study had significantly lower natural protein intake compared with those without MBD, and the sapropterin dihydrochloride-treated individuals who were able to relax restrictions to natural protein intake in this study did not develop MBD, suggesting that a decrease in natural protein intake has a role in the development of MBD [68].

Two studies identified in our SLR investigated the nutritional status of individuals with different PKU phenotypes [45, 69]. One study [69] reported significantly lower concentrations of selenium in those with PKU receiving AAM compared with those with HPA; the authors suggested that this result was affected by adults relaxing restrictions to a low-protein diet and, hence, overall intake of amino acids may be higher than the prescribed AAM. Another study [45] also found significantly lower selenium and phosphorus concentrations in those with PKU versus those with HPA but concluded that this was related to increased dietary adherence and younger age (< 18 years). Conversely, serum pre-albumin, ferritin, and folic acid concentrations were significantly reduced in those with mild HPA versus those with PKU and vitamin B12 and zinc were significantly reduced in those with mild HPA and mild or moderate PKU compared with those with cPKU [45]. The positive correlation of vitamin B12 and zinc concentrations with disease severity was surprising but the authors postulated that despite lower intake of these nutrients through natural protein sources in those with cPKU, the cobalamin content of Phe-free amino acid supplements is high and there is a more efficient absorption of zinc salts from supplements than from meals [45].

The relationship between PKU severity and the risk of overweight/obesity is unclear. Mazzola et al. [50] found no effect of PKU phenotype on BMI classification or bioelectrical impedance parameters, whereas Couce et al. [44] reported overweight/obesity and worse insulin resistance (a marker of the metabolic syndrome) in a significantly higher proportion of individuals with PKU versus mild HPA and healthy controls, particularly in those with a late diagnosis. Higher levels of insulin resistance were correlated with increasing BMI, WC, and age [44]; however, in a linear regression model, age had the most influence on BMI; therefore, the authors concluded that the cause of increased insulin resistance in PKU is likely to be multifactorial [44].

Due to the small number of studies included in this population comparison and the breadth of outcome measures reported, it is not feasible to draw definitive conclusions on the impact of disease severity on the clinical burden of somatic comorbidities.

### Clinical implications

The higher clinical burden of somatic comorbidities in individuals with PKU compared with a general (non-PKU) population, as reported here and in other studies, contributes to the clinical and socioeconomic burden of PKU; higher rates of prescribed treatment use have been reported, and mean healthcare costs are significantly greater (*p* < 0.0001) than for the general population [15, 16]. The findings from this review point to an unmet need for optimized approaches to Phe control, to maintain Phe levels within recommended ranges over the long term and potentially avoid somatic comorbidities and the associated clinical and socioeconomic implications.

Adherence to dietary restrictions can be challenging, especially as individuals with PKU progress to adulthood. Limited and unpleasant food choices, as well as the overall inconvenience and time-consuming nature of a restricted diet and effect on socializing, are cited as factors negatively impacting adherence in adults with PKU [81, 82]. Patients and their caregivers report a lack of disease awareness in hospitality settings, leading to concern around menu choices outside the home that are incompatible with dietary restrictions [83]. The challenge of dietary adherence may impact a patient’s quality of life (QoL). Studies using a PKU-specific questionnaire to evaluate the effect of PKU on QoL [84, 85], reported the emotional impact of PKU and the management of PKU (anxiety about blood Phe levels; guilt regarding poor adherence to dietary restrictions) as high scoring questionnaire domains, indicating a negative impact on QoL. The unpleasant taste of food supplements was also considered a main issue [85]. Another study [86], using preference-based measures to estimate the effect of PKU on health-related QoL, reported dietary restrictions and symptoms of PKU as both having a significant negative impact on health-related QoL.

There is a need for careful monitoring of nutritional intake as a component of nutritional status assessment, independent of treatment modality. Closer medical surveillance may prevent losing individuals to follow-up, particularly as they progress from adolescence to adulthood, and increased awareness of screening for comorbidities may identify individuals in most need of improved Phe control. It is important that individuals with PKU, who already have limited options in food choices owing to their Phe-restricted diet, receive ongoing personalized nutritional counselling, with methodical nutritional status monitoring from a multidisciplinary team specialized in inherited metabolic disorders to prevent overweight, obesity, and its related comorbidities.

Some treatment approaches may improve Phe and natural protein tolerance, reducing the reliance on strict Phe restriction for metabolic control, which may lead to improvements in QoL over time. Although a meta-analysis of outcomes before and after relaxation of a Phe-restricted diet with sapropterin dihydrochloride did not find an improvement in QoL following treatment, the authors of the study noted this finding does not reflect clinical practice [87]. One explanation offered for this anomaly was that the general QoL questionnaires used in the majority of studies included in the analysis may not be sensitive enough to capture the daily burden of a highly restricted diet [87]. Indeed, the only study utilizing a PKU-specific QoL questionnaire to investigate the impact of treatment on QoL, reported significant improvements in self-reported impact and satisfaction sub-scores and total QoL score over time in adolescents and adults responding to sapropterin dihydrochloride, and QoL improvements were associated with increased Phe tolerance [88]. It is important to note that these improvements can only be achieved when an adequate and balanced diet is also maintained. Educating both clinicians and individuals with PKU on the role of balanced nutrition to effectively manage and/or prevent chronic disease is required [89, 90], as well as adjusting nutrition according to pharmacologic intervention.

### Strengths and limitations of the review methodology

This review involved sensitive searches of the peer-reviewed literature only and was guided by the pre-defined eligibility criteria established in the protocol. Comprehensive, relevant, and accurate data abstraction was ensured throughout the review process.

Strengths of this review include the consideration of any somatic comorbidity in assessing clinical burden, and the focus primarily on an adult population (studies conducted exclusively in children and adolescents were excluded). With the introduction of PKU testing into newborn screening programs more than half a century ago, and the availability of treatment accordingly, the global PKU population is increasing in age. The neuropsychologic burden of PKU is well documented, particularly in children, and this review provides new insight into the somatic comorbidity burden in adults.

Heterogeneity in the studies that were included in this review, in terms of the clinical outcomes used (and their definitions); how each outcome was measured; and study designs, including interventions and comparators, meant meta-analysis of effect estimates was not considered appropriate for the majority of outcomes. Vote counting is considered an acceptable alternative synthesis method when meta-analysis is not feasible [21], and an assessment of the direction of effect of comorbidity burden was still able to be made, even when several studies did not report statistical significance of the difference between groups. Hence, the results of the vote-counting analysis provided an overview of the somatic comorbidity burden, in terms of both the range of comorbidities and the direction of effect, in individuals with PKU compared with a non-PKU population. However, vote counting does have limitations: the analysis does not account for differences in the relative sizes of the studies or methodological aspects and provides no information on the magnitude of effect; it is also difficult to interpret the results when studies report multiple outcomes and/or measures and the direction of effect differs between them. This is compounded by inclusion of small numbers of studies, as was the case for the comparison between individuals with PKU with different levels of adherence to a Phe-restricted diet. Other alternative synthesis methods to meta-analysis are more powerful than vote counting (e.g., combining *p* values), but were not appropriate for this review due to incomplete data. Meta-analysis was only feasible for BMD Z-scores from a sub-set of the studies reporting bone-related abnormalities included in the SLR (see Fig. 4). The objectives of the meta-analysis were more specific than those of the broader SLR and therefore are reported separately.

It is important to acknowledge that the grouping of individual somatic comorbidities into comorbidity types could have been done differently and may have resulted in different conclusions being drawn from the data. In this study, cardiovascular outcomes were among the most reported somatic comorbidities with a higher burden in individuals with PKU on a Phe-restricted diet with or without pharmacologic therapy (*n* = 5). The grouping of cardiovascular outcomes with other comorbidities that are considered risk factors for cardiovascular disease (e.g., diabetes, overweight/obesity, hypertension) was explored, but not undertaken due to variance in opinion on the most appropriate categorization given the complexities of these individual comorbidities, and to maintain a level of granularity on the comorbidity burden in PKU. Consequently, the overall cardiovascular burden in individuals with PKU in the current treatment landscape may be underestimated in the vote-counting analysis.

This review considered studies published in English and retrievable via the PubMed® interface, and in-built filters for human studies and adult age-groups were employed, which rely on appropriate indexing within the MEDLINE database for retrieval. MEDLINE has relatively broad coverage of the medical literature and it is likely that the majority of relevant studies would have been identified, but it is important to acknowledge that any studies reported outside of MEDLINE and other sources accessible via PubMed®, or outside the filters employed in the search, will have been missed.

Finally, it is important to note that a formal risk of bias assessment of the studies included in this review was not conducted, limiting the certainty of the findings. There was a high degree of heterogeneity across studies in terms of different study designs, outcomes and outcome measures reported, patient characteristics, and components of MNT. As such, there is a clear high-risk of bias rendering a formal assessment as unnecessary. However, despite the potential for a loss of the ‘signal’ relating to a higher burden of somatic comorbidities in individuals with PKU compared with healthy controls, owing to the heterogeneity across studies, the results of the vote-counting analysis did show a consistent direction of effect. It is important to acknowledge that there is a potential for publication bias. A formal assessment of publication bias would be challenging for the spread of outcomes and measures reported in this SLR. However, it is possible that studies that did not report a higher burden of somatic comorbidities in individuals with PKU have not been published, which would mean that the higher burden in individuals with PKU may be overestimated. Despite this, the results of the vote-counting analysis were consistently in favor of a higher burden in individuals with PKU versus healthy controls. The potential for publication bias to overestimate the proportion of studies showing a higher burden in individuals with PKU versus healthy controls was minimized by excluding single-cohort studies in which there was no comparison between individuals with PKU and healthy controls or reference values. We believe that this supports our conclusions of a higher somatic comorbidity burden in individuals with PKU, with the caveat that vote-counting analysis is associated with limitations.

### Strengths and limitations of the included evidence

Studies included in this review represented individuals with PKU across Asia, Europe, North America, and South America, although most studies were conducted in Europe or the United States, suggesting findings will be broadly generalizable to these region-specific PKU populations. There was a broad publication date range for the included studies, spanning from 1990 to 2023 as no date restrictions were applied to the search. However, improvements in the taste and palatability of protein substitutes and low protein foods that have occurred over time could have improved patient compliance, complicating the interpretation of results across older and newer studies. Most studies identified for inclusion in the review were observational in design, supporting the need for systematic assessments of data such as this one.

Although one study [22] compared results for the overall population with PKU and results for a subgroup of individuals with early diagnosed PKU against results for the control population and another study [50] stated that time of diagnosis did not affect anthropometric or bioelectrical impedance parameters (such that results from all individuals with PKU were analyzed as a single group), only two studies included in the review directly compared the somatic comorbidity burden in early and late-diagnosed adults with PKU [15, 44], making it difficult to further evaluate comorbidity burden by time of diagnosis or period of treatment. In the claims-based study that was identified and included [15], a range of comorbidity types was reported in early- and late-diagnosed adults with PKU and at a higher prevalence than a matched control population for many, including infectious gastroenteritis and colitis, overweight and obesity, hypotension, and disorders of lipid metabolism and other lipidemias. In a cross-sectional study conducted in a Spanish PKU population [44], there was a higher proportion of those with BMI above the upper limit in late-diagnosed compared with early-diagnosed individuals but this was found to be largely driven by the effect of age.

Seven studies included in the comparison of comorbidity burden in a Phe-restricted diet population versus healthy controls or reference values were conducted in mixed treatment populations [15, 22, 24, 25, 32, 46, 52]. These studies included some individuals on pharmacologic therapy in addition to a Phe-restricted diet [15, 22, 25, 32, 52, 57], and individuals on and not on a Phe-restricted diet [24, 52]. These studies were all included but results were not stratified by treatment type. A large proportion of studies included in the SLR did not stratify results by age-groups such that participants < 16 years of age could have been included; the relevance of this to the findings of the review is unknown. The scarcity of data from studies using similar study designs and patient populations, as well as consistency in outcomes used and the way they are measured for many comorbidities, restricts the synthesis methods that can be used to evaluate the burden of somatic comorbidities in adults with PKU. Although vote counting is considered an acceptable alternative synthesis method when meta-analysis is not feasible [21], not all studies could be included in the vote-counting analysis reported here due to lack of comparative data. A lack of reporting of statistical significance of differences between PKU populations in individual studies also limited interpretation of the presence or absence of a difference in comorbidity burden.

### Future studies

The variety of outcomes reported, and outcome measures used in studies included in this review, limited the synthesis of data across many somatic comorbidity types and PKU populations and, accordingly, the conclusions that can be drawn. There is a need for studies assessing somatic comorbidities using more robust study designs and consistent outcome measures, and including specific PKU population comparisons (e.g., by disease severity, timing of diagnosis and treatment [early or late], adherence to treatment, male:female ratio, and age). Consideration of the aging PKU population will be important, given older adults may have different comorbidities to a younger adult population.

It is unclear whether the somatic comorbidity burden in PKU comes from the disease itself or from adherence to severe dietary restrictions and/or inadequate supplementation of amino acids and micronutrients. There is a need to further evaluate the relationship between effective metabolic control and comorbidity burden to understand whether control of blood Phe levels can reduce the incidence of such complications. Consideration of the multidisciplinary healthcare team structure will be important, as adherence to diet may be lower in those centers where individuals are managed by an incomplete team, particularly for individuals with cPKU.

Studies investigating the burden of illness of PKU, incorporating both patient and caregiver health-related QoL assessed with disease-specific instruments, and the impact of different treatments, will provide insight into the indirect costs of somatic comorbidities in individuals with PKU, furthering understanding of the socioeconomic implications of somatic comorbidities and impact of effective treatment. Pessoa et al. also highlighted the need for burden of illness studies to identify the range of ongoing and significant complications experienced by individuals with PKU in order to inform healthcare providers and public health authorities [70].

## Conclusions

Individuals with PKU have a higher somatic comorbidity burden versus a non-PKU population, highlighting the unmet need for optimized approaches to blood Phe control in this population. Improved access to therapeutic interventions to maintain blood Phe levels within recommended ranges over the long term may potentially avoid the clinical and economic implications of managing comorbidities. To build on the evidence from this review and better understand the relationship between blood Phe control, adherence to diet and comorbidity burden, more robust studies reporting consistent outcome measures are needed, especially in specific PKU populations.

### Supplementary Information

**Additional file 1: Table S1.** PubMed® search string.

**Additional file 2: Figure S1.** Distribution of studies by study design.

**Additional file 3: Figure S2.** Distribution of studies by geographic location.

## Data Availability

The datasets and study protocol used and/or analyzed during the current study are available from the corresponding author on reasonable request. Additional supporting documents may be available upon request. Investigators will be able to request access to these data and supporting documents via a data sharing portal (https://www.biomarin.com/our-science/funding-and-support/publication-data-request/) beginning 6 months and ending 2 years after publication. Data associated with any ongoing development program will be made available within 6 months after approval of relevant product. Requests must include a research proposal clarifying how the data will be used, including proposed analysis methodology. Research proposals will be evaluated relative to publicly available criteria available at https://www.biomarin.com/our-science/funding-and-support/publication-data-request/ to determine if access will be given, contingent upon execution of a data access agreement with BioMarin Pharmaceutical Inc.

## Abbreviations

AAM: Amino acid mixture
AV: Assigned value
BAP: Bone-specific alkaline phosphatase
BCM: Body cell mass
BH4: Tetrahydrobiopterin
BMD: Bone mineral density
BMI: Body mass index
Ca/Cr: Calcium/creatinine index
CCI: Charlson Comorbidity Index
CGMP-AA: Modified casein glycomacropeptide amino acid
CI: Confidence interval
CIMT: Carotid intima media thickness
COPD: Chronic obstructive pulmonary disease
cPKU: Classical PKU
DHA: Docosahexaenoic acid
ECM: Extracellular mass
EPA: Eicosapentaenoic acid
GERD: Gastroesophageal reflux disease
GMP: Glycomacropeptide-based protein
GMP-AA: Glycomacropeptide-based protein amino acid
HOMA-IR: Homeostasis model assessment insulin resistance
HPA: Hyperphenylalaninemia
IQR: Interquartile range
L-AA: L-amino acid
L-AAM: L-amino acid mixture
LBM: Lean bone mass
LNAA: Large neutral amino acids
LAT1: L-type amino acid transporter 1
MBD: Mineral bone disease
MeSH: Medical Subject Headings
MMA: Methylmalonic acid
MNT: Medical nutrition therapy
NHANES: National Health and Nutrition Examination Survey
NR: Not reported
OC: Osteoclastogenesis
PAH: Phenylalanine hydroxylase
Phe: Phenylalanine
PICOS: Population, Intervention, Comparator, Outcome, Study design
PKU: Phenylketonuria
PR: Prevalence ratio
PRISMA: Preferred Reporting Items for Systematic Reviews and Meta-Analyses
PS: Protein substitute
QoL: Quality of life
SBMD: Spongy bone mineral density
SD: Standard deviation
SE: Standard error
SIRS: Systemic inflammatory response syndrome
SLR: Systematic literature review
SSI: Strength strain index
SWiM: Synthesis Without Meta-analysis
T4: Thyroxine
TBMD: Total bone mineral density
TSH: Thyroid-stimulating hormone
UIC: Urinary iodine concentration
UIC/Cr: Creatinine-normalized urinary iodine concentration
WC: Waist circumference
