# Supplementary material for: Systematic literature review of the somatic comorbidities experienced by adults with phenylketonuria
Source: Orphanet J Rare Dis. 2024 Aug 12;19:293. doi: 10.1186/s13023-024-03203-z (PMC11318169; doi:10.1186/s13023-024-03203-z)
Supplement: Supplementary file 1 — Additional file 1: Table S1. PubMed® search string. [file 13023_2024_3203_MOESM1_ESM.docx]

Additional file 1: Table S1 PubMed^®^ search string

| **Search No.** | **Query** | **Details** | **No. of hits** |
| --- | --- | --- | --- |
| 1 | Disease | “Phenylketonuria”[Title/Abstract] OR “phenylketonuri*”[Title/Abstract] OR “phenylalanine hydroxylase deficien*”[Title/Abstract] OR “phenylalanine deficien*”[Title/Abstract] OR “phenylalanine deficiency syndrome”[Title/Abstract] OR “hyperphenylalanin*”[Title/Abstract] OR “hyper-phenylalanin*”[Title/Abstract] OR “PKU”[Title/Abstract] |  |
| 2 | Disease burden/ comorbidities | "Disease Burden"[Title/Abstract] OR "burden of disease"[Title/Abstract] OR "comorbid*"[Title/Abstract] OR "co-morbid*"[Title/Abstract] OR "complication*"[Title/Abstract] OR "Burden of illness"[Title/Abstract] OR "Illness Burden"[Title/Abstract] OR “somatic comorbid*”[Title/Abstract] OR “somatic co-morbid*”[Title/Abstract] |  |
| 4 | HRQoL burden | "quality of life"[MeSH Terms] OR "health-related quality of life"[Text Word] OR "health related quality of life"[Text Word] OR "HRQOL"[Text Word] OR "quality of life"[Text Word] OR "Life quality"[Text Word] OR "health status"[Text Word] OR "Sickness Impact Profile"[MeSH Terms] OR "Activities of Daily Living"[MeSH Terms] OR "medical outcomes survey"[Text Word] OR "disabilit*"[Text Word] OR "short form 36"[Text Word] OR "SF-36"[Text Word] OR “EQ-5D”[Text Word] OR “social isolation”[MeSH Terms] OR “social exclusion*”[Text Word] OR “ostracis*”[Text Word] OR “social impact”[Text Word] OR “social behavior”[Text Word] OR “social behaviour”[Text Word] OR “socio-occupational”[Text Word] OR “socio occupational” [Text Word] OR “social function*”[Text Word] OR “Stress Disorders, Traumatic, Acute”[MeSH Terms] OR “stress”[Title/Abstract] OR “stress”[Text Word] “chronic stress”[Text Word] OR “Oxidative stress”[MeSH Terms] |  |
| 5 | Bone density | (“bone density”[Title/Abstract]) OR (“bone mineral density”[Title/Abstract]) OR (“Bone Density”[MesH Terms]) OR (“bone mineral densit*”[Text Word]) OR (“bone mineral content*”[Text Word]) OR (“Osteoporosis”[MeSH Terms]) OR (“post-traumatic osteoporos*”[Text Word]) OR (“chronic osteoporosis”[Text Word]) OR (“bone loss”[Text Word]) OR (“bone-loss”[Text Word]) |  |
| 6 | Cardiovascular disease | (“Risk of cardiovascular”[Title/Abstract]) OR (“Cardiovascular*”[Title/Abstract]) (Heart Diseases[MeSH Terms]) OR (Heart Disease*[Text Word])) OR (Cardiac Disease*[Text Word])) OR (Cardiac Disorder*[Text Word])) OR (Heart Disorder*[Text Word])) OR (Myocardial Infarction[MeSH Terms])) OR (Myocardial Infarction*[Text Word])) OR (heart attack*[Text Word])) OR ("Myocardial Infarct"[Text Word])) ) OR (Heart Failure[MeSH Terms])) OR ("heart failure"[Text Word])) OR ("Cardiac Failure"[Text Word])) OR ("Myocardial Failure"[Text Word])) OR (Cardiomyopathies[MeSH Terms])) OR (Cardiomyopath*[Text Word])) OR (myocardial disease*[Text Word])) OR (cardiac complication*[Text Word])) OR (heart complication*[Text Word])) OR (myocardial complication*[Text Word]) |  |
| 7 | Gastrointestinal disorders | (“Gastrointestinal”[Title/Abstract]) (“Gastro-intestinal”[Title/Abstract]) OR (Gastrointestinal Diseases[MeSH Terms]) OR (gastrointestinal disorder*[Text Word]) (gastro-intestinal disorder*[Text Word]) OR (functional gastrointestinal disorder*[Text Word]) OR (functional gastro-intestinal disorder*[Text Word]) |  |
| 8 | Dermatologic disorders | (“Dermatologic*”[Title/Abstract]) OR (Skin Diseases[MeSH terms]) OR (Atopic dermatitis[Text Word]) OR (Dermatitis[Text Word]) (skin tissue disorder*[Text Word]) OR (subcutaneous tissue disorder*[Text Word]) |  |
| 9 | Obesity/ overweight | (“obes*”[Title/Abstract]) OR (“overweight”[Title/Abstract]) OR (“BMI”[Title/Abstract]) OR (“body mass index”[Title/Abstract]) OR (Obesity[MeSH Terms]) OR (body weight[Text Word]) OR (Overweight[MeSH Terms]) |  |
| 10 | Diabetes | (“Diabet*”[Title/Abstract]) OR (“Insulin”[Title/Abstract]) OR (Diabetes Mellitus [MeSH Terms]) OR (anti diabet*[Text Word]) OR (anti-diabet*[Text Word]) |  |
| 11 | Migraine/ headache | (“Migraine*”[Title/Abstract]) OR (“headache*”[Title/Abstract]) OR (Headache Disorders[MeSH Terms]) OR (Migraine Disorders[MeSH Terms]) OR (headache*[Text Word]) OR (migraine[Text Word]) |  |
| 12 | COPD/ asthma | (“COPD”[Title/Abstract]) OR (“chronic obstructive pulmonary disease”[Title/Abstract]) OR (“pulmonary disease*”[Title/Abstract]) OR (“lung disease”[Title/Abstract]) OR (“asthma”[Title/Abstract]) OR (Pulmonary Disease, Chronic Obstructive[MeSH Terms]) OR (Chronic Obstructive Lung Disease*[Text Word]) OR (Chronic Obstructive Pulmonary Disease*[Text Word]) OR (air flow obstruction*[Text Word]) OR (Chronic airflow obstruction*[Text Word]) OR (COPD[Text Word]) OR (COAD[Text Word]) OR (Asthma[MeSH Terms]) OR (asthma*[Text Word]) OR (bronchial asthma*[Text Word]) OR (chronic obstructive airway disease[Text Word]) |  |
| 13 | Cancer | (“cancer”[Title/Abstract]) OR (“neoplasm*”[Title/Abstract]) OR (Neoplasms[MeSH Terms) OR (neoplasia*[Text Word]) OR (Neoplasm*[Text Word]) OR (Tumor*[Text Word]) OR (Tumour[Text Word]) OR (cancer*[Text Word]) OR (malignanc*[Text Word]) |  |
| 14 | Neurologic disorders | (“neurologic*”[Title/Abstract]) OR (Nervous System Diseases[MeSH Terms) OR (Neurologic Disorder*[Text Word]) OR (Neurological Disorder*[Text Word]) OR (Nervous System Disorder*[Text Word]) OR (“tremor”[Text Word]) |  |
| 15 | Sleep disorders | (“Sleep Wake Disorders”[MeSH Terms]) OR (“sleep disorder*”[Text Word]) OR (“neurogenic tachypnea”[Text Word]) OR (“sleeper syndrome”[Text Word]) OR (“short sleep”[Text Word]) OR (“sleep disturb*”[Text Word]) OR (“insomnia”[Text Word]) OR (“wakefulness”[Text Word]) OR (“disturbed sleep”[Text Word]) OR (“sleep”[Text Word]) OR (“sleep pattern”[Text Word]) |  |
| 16 | Nutritional disorders | (“feeding and eating disorders”[MeSH Terms]) OR (“abnormal eating”[Text Word]) OR (“eating disorder”[Text Word]) OR (“nutritional disorder*”[Text Word]) OR (“nutrient deficienc*”[Text Word]) OR (“nutritional status”[Text Word]) OR (“protein consumption”[Text Word]) OR (“protein tolerance”[Text Word]) OR (“protein intolerance”[Text Word]) OR (“nutritional deficienc*”[Text Word]) OR (“diet*”[Text Word]) OR (“dietary composition”[Text Word]) OR (“disordered eating”[Text Word]) |  |
| 17 |  | 1 AND (2 OR 3 OR 4 OR 5 OR 6 OR 7 OR 8 OR 9 OR 10 OR 11 OR 12 OR 13 OR 14 OR 15 OR 16) | 6479 |
| 18 |  | ("Editorial"[Publication Type] OR "Letter"[Publication Type] OR "Comment"[Publication Type]) |  |
| 19 | Exclude unwanted study types | 17 NOT 18 | 6239 |
| 20 | Limit to studies on adults | 19 AND adult age filters | 1256 |
| 21 | Limit to human studies published in English | 20 AND Human studies filter AND English language filter | 1128 |

Results column shows results from the original search conducted on February 1, 2022.

COAD, chronic obstructive airway disease; COPD, chronic obstructive pulmonary disease; HRQoL, health-related quality of life; MeSH, medical subject headings; PKU, phenylketonuria
