# Supplementary material for: Systematic literature review of the somatic comorbidities experienced by adults with phenylketonuria
Source: Orphanet J Rare Dis. 2024 Aug 12;19:293. doi: 10.1186/s13023-024-03203-z (PMC11318169; doi:10.1186/s13023-024-03203-z)
Supplement: Supplementary file 2 — Additional file 2: Figure S1. Distribution of studies by study design. [file 13023_2024_3203_MOESM2_ESM.docx]

Additional file 2: Figure S1 Distribution of studies by study design
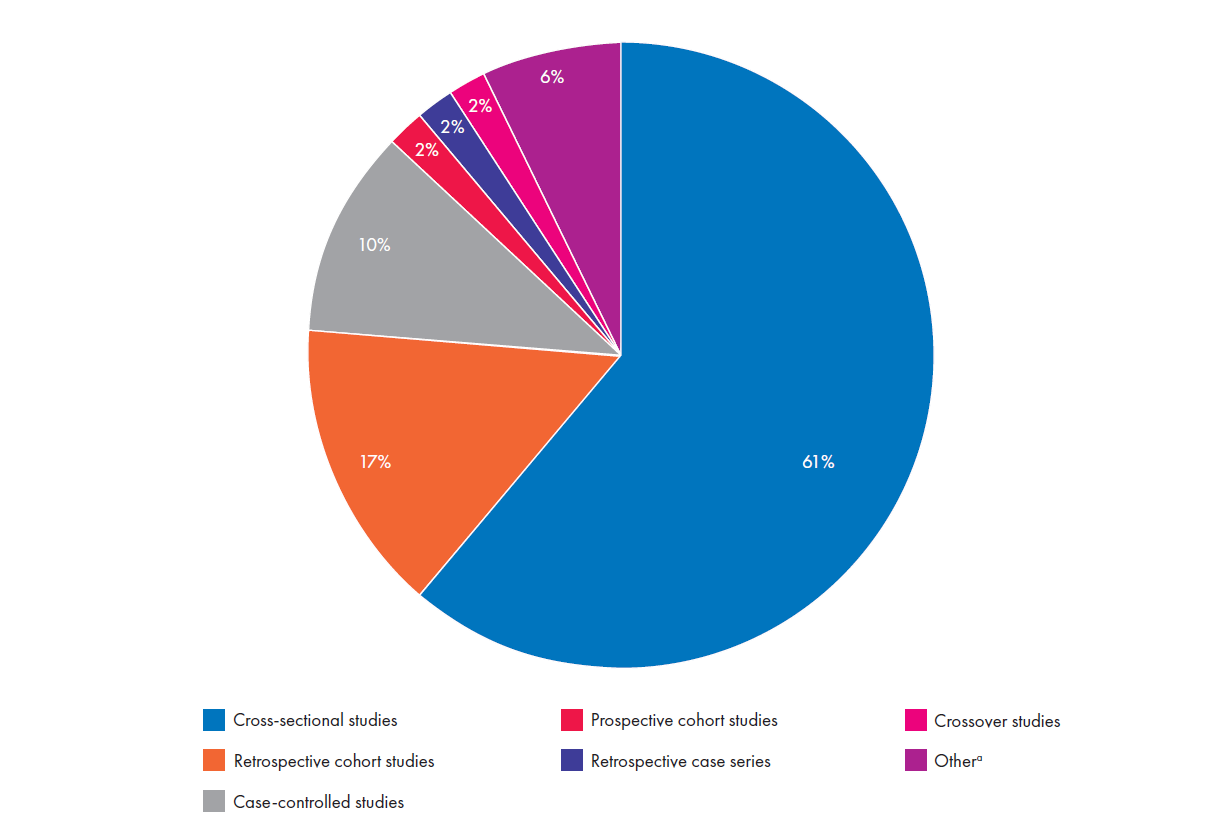


^a^Includes open interventional trials, pooled analyses, and cost analyses
