# Supplementary material for: Systematic literature review of the somatic comorbidities experienced by adults with phenylketonuria
Source: Orphanet J Rare Dis. 2024 Aug 12;19:293. doi: 10.1186/s13023-024-03203-z (PMC11318169; doi:10.1186/s13023-024-03203-z)
Supplement: Supplementary file 3 — Additional file 3: Figure S2. Distribution of studies by geographic location. [file 13023_2024_3203_MOESM3_ESM.docx]

Additional file 3: Figure S2 Distribution of studies by geographic location

Where a study has been conducted in more than one country, that study has been included in the count for each country
